# Carriage of Shiga toxin phage profoundly affects *Escherichia coli* gene expression and carbon source utilization

**DOI:** 10.1186/s12864-019-5892-x

**Published:** 2019-06-17

**Authors:** Petya Berger, Ivan U. Kouzel, Michael Berger, Nadja Haarmann, Ulrich Dobrindt, Gerald B. Koudelka, Alexander Mellmann

**Affiliations:** 1 Institute of Hygiene, University of Münster, Münster, Germany; 2 Institute of Bioinformatics, University of Münster, Münster, Germany; 3 Department of Biological Sciences, University at Buffalo, Buffalo, USA

**Keywords:** Stx2a phage, *E. coli* O104:H4, *E. coli* O157:H7, *E. coli* K-12 MG1655, RNA-seq, Biolog phenotype microarrays, Carbon source utilization, Carbon metabolism

## Abstract

**Background:**

Enterohemorrhagic *Escherichia coli* (*E. coli*) are intestinal pathogenic bacteria that cause life-threatening disease in humans. Their cardinal virulence factor is Shiga toxin (Stx), which is encoded on lambdoid phages integrated in the chromosome. Stx phages can infect and lysogenize susceptible bacteria, thus either increasing the virulence of already pathogenic bacterial hosts or transforming commensal strains into potential pathogens. There is increasing evidence that Stx phage-encoded factors adaptively regulate bacterial host gene expression. Here, we investigated the effects of Stx phage carriage in *E. coli* K-12 strain MG1655. We compared the transcriptome and phenotype of naive MG1655 and two lysogens carrying closely related Stx2a phages: ϕO104 from the exceptionally pathogenic 2011 *E. coli* O104:H4 outbreak strain and ϕPA8 from an *E. coli* O157:H7 isolate.

**Results:**

Analysis of quantitative RNA sequencing results showed that, in comparison to naive MG1655, genes involved in mixed acid fermentation were upregulated, while genes encoding NADH dehydrogenase I, TCA cycle enzymes and proteins involved in the transport and assimilation of carbon sources were downregulated in MG1655::ϕO104 and MG1655::ϕPA8. The majority of the changes in gene expression were found associated with the corresponding phenotypes. Notably, the Stx2a phage lysogens displayed moderate to severe growth defects in minimal medium supplemented with single carbon sources, e.g. galactose, ribose, L-lactate. In addition, in phenotype microarray assays, the Stx2a phage lysogens were characterized by a significant decrease in the cell respiration with gluconeogenic substrates such as amino acids, nucleosides, carboxylic and dicarboxylic acids. In contrast, MG1655::ϕO104 and MG1655::ϕPA8 displayed enhanced respiration with several sugar components of the intestinal mucus, e.g. arabinose, fucose, N-acetyl-D-glucosamine. We also found that prophage-encoded factors distinct from CI and Cro were responsible for the carbon utilization phenotypes of the Stx2a phage lysogens.

**Conclusions:**

Our study reveals a profound impact of the Stx phage carriage on *E. coli* carbon source utilization. The Stx2a prophage appears to reprogram the carbon metabolism of its bacterial host by turning down aerobic metabolism in favour of mixed acid fermentation.

**Electronic supplementary material:**

The online version of this article (10.1186/s12864-019-5892-x) contains supplementary material, which is available to authorized users.

## Background

Enterohemorrhagic *E. coli* (EHEC) are intestinal pathogenic bacteria, which cause diarrhea, hemorrhagic colitis (HC, bloody diarrhea) and the potentially fatal hemolytic uremic syndrome (HUS). HUS is characterized by the triad of hemolytic anemia, thrombocytopenia and acute kidney injury [1]. Due to its ability to cause HUS, EHEC is the most frequent cause of renal failure in children [2].The majority of EHEC-associated HC and HUS have been attributed to the *E. coli* serotype O157:H7, although non-O157 serotypes are increasingly recognized as being clinically important [1, 3–5].

The hallmark of an EHEC infection is the production of Shiga toxins (Stxs).These toxins are AB_5_ toxins that irreversibly inhibit host cell protein synthesis and lead to cell death [6]. There are two immunologically distinct Stx types, Stx1 and Stx2, which are further divided into the subtypes Stx1a,c,d and Stx2a-i [7–9], with Stx2a being the one most often associated with severe illness and the development of HUS [10]. Shiga toxins are encoded by prophages that are resident in EHEC and all EHEC strains harbor at least one Stx-encoding bacteriophage. The Stx-encoding phages are lysogenic converting λ-like phages. Stx phages are very sequence heterogeneous, however their overall genome organization, gene regulatory patterns and lifecycle are similar to those of non-Stx-encoding λ-like bacteriophages. Upon infecting a bacterial host, a lambdoid phage chooses between two developmental fates. The phage can grow lytically, thereby killing the host. Alternatively, in lysogenic growth the phage chromosome inserts into the host genome where, under the control of the DNA binding phage CI repressor, the prophage genome lies essentially dormant. Upon activation of the SOS response, a RecA-mediated cleavage of the CI repressor causes its dissociation from the DNA and thus triggers lytic growth. Induction/lytic phage growth is essential to EHEC pathogenesis because high level Stx production and its subsequent release only occurs during phage lytic growth [11–16].

Stx phages are highly mobile elements that mediate the horizontal gene transfer of *stx* genes and therefore contribute to the emergence of new pathotypes [17]. *E. coli* O104:H4, which caused in 2011 a massive EHEC outbreak in Germany shares highest genome sequence similarity with enteroaggregative *E. coli* (EAEC) and does not carry the locus of enterocyte effacement (LEE) pathogenicity island characteristic of typical EHEC isolates [18]. This led to the hypothesis that the 2011 outbreak strain has originated via Stx2a phage acquisition by an EAEC ancestor [19, 20]. Similarly, the EHEC strain *E. coli* O157:H7 is believed to have evolved from enteropathogenic *E. coli* through a series of horizontal gene transfer events including the acquisition of Stx1a- and/or Stx2a-encoding phages [21, 22]. Stx2 phages are able to infect and lysogenize both pathogenic and nonpathogenic enteric *E. coli* isolates in vitro, even if the hosts already possessed a Stx phage. These new lysogens can produce infectious phage particles [23]. Moreover, successful in vivo transduction of susceptible bacteria with both Stx1 and Stx2 phage derivatives has been reported [24, 25] and newly created lysogens of commensal *E. coli* can produce high levels of Stx2 [26, 27]. In a mouse model infected with *E. coli* O157:H7, Stx is more often detected in the feces of mice co-colonized with Stx phage-sensitive than with phage-resistant *E. coli.* This finding suggests that the commensal *E. coli* can function as surrogate hosts for Stx phage, leading to increased Stx production [28]. Thus Stx phage-susceptible commensal bacteria apparently can substantially enhance the severity of an EHEC infection.

There is increasing evidence that Stx prophage-encoded factors can modify host gene expression and thereby its phenotype. For example, microarray analysis has revealed that the Stx2a phage ϕMin27 lysogeny in *E. coli* K-12 strain MG1655 (MG1655) leads to the differential expression of more than 150 bacterial host genes and enhances its acid resistance and motility [29]. Similarly, transcriptomic studies applying RNA sequencing (RNA-seq) has revealed a positive effect of the Stx2a phage ϕ24_B_ carriage on the expression of acid resistance genes in the *E. coli* K-12 strain MC1061. This effect is mediated by the phage-encoded regulator CII [30]. Moreover, CII also apparently represses expression of elements of the LEE-encoded type III secretion system, which is crucial for EHEC virulence [31]. Also, the phage encoded anti-repressor Cro, which mediates the switch to the phage lytic cycle, activates LEE gene expression in EHEC 8624. In addition, Cro apparently affects the expression of nearly 900 genes in this strain [32].

Here, we investigated the effects of the Stx phage carriage on host gene expression and phenotype. For this work we examined the effects of two different Stx2a prophages on the commensal strain MG1655. The Stx2a-encoding phages were derived from the exceptionally pathogenic 2011 *E. coli* O104:H4 outbreak strain and the *E. coli* O157:H7 strain PA8. Despite being found in two different EHEC serotypes, these two phages have a high degree of sequence similarity [33]. Naive MG1655 and the Stx2a phage converted MG1655::ϕO104 and MG1655::ϕPA8 were subjected to quantitative RNA-seq analysis and phenotype analysis including microarrays. We found that the prophages had a profound impact on MG1655 host gene expression, which was in turn resulting in stable, dramatic changes in the carbon source utilization.

## Results

### Transcriptome analysis of naive MG1655 and the Stx2a phage lysogens MG1655::ϕO104 and MG1655::ϕPA8

**Fig. 1:**
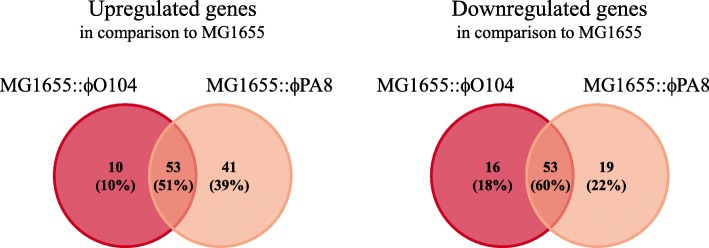
Overlap of genes found to be differentially expressed in the Stx2a phage lysogens in comparison to naive MG1655. The Venn diagram shows the overlap of genes found up- or downregulated in MG1655::ϕO104 and MG1655::ϕPA8

In order to analyze the influence of Stx phage carriage on host cell transcription, we performed strand specific RNA-seq with total RNA isolated from exponential phase naive MG1655 and the Stx2a phage lysogens MG1655::ϕO104 and MG1655::ϕPA8 grown under standard laboratory conditions (see Methods). On average, 14 million reads (range, 10–19 million reads) were obtained per library and more than 94% of the reads in each dataset were mapped to the respective reference genomes (Additional file 3: Table S1). Principal component analysis based on the gene expression data quantified with DESeq2 showed little variation between the biological triplicates within a strain; however there was a clear difference in between the analyzed strains (Additional file 3: Figure S1). The DESeq2 analysis further revealed that both Stx2a phage lysogens displayed multiple examples of differential gene expression in comparison to the naive MG1655 (Additional file 1 and Additional file 2). Using an adjusted *p*-value of < 0.1 and a cut-off of 1.5-fold difference in the expression, we found that a total of 63 genes were significantly upregulated and 69 genes were downregulated in MG1655::ϕO104. Similarly, we found 94 upregulated and 72 downregulated genes in MG1655::ϕPA8 (Additional file 3: Tables S2 and S3). Importantly, we found that there was a substantial overlap of the genes that were differentially expressed in MG1655::ϕO104 and MG1655::ϕPA8 in comparison to naive MG1655. Our analysis indicated that 51% of the up- and 60% of the downregulated genes were shared between the lysogens (Fig. 1). In addition, 40–79% of the lysogen-specific genes were accordingly up- or downregulated in the other strain, however below one of the thresholds applied in our analysis (Additional file 1, Additional file 2 and Additional file 3: Tables S2 and S3). This observation was in agreement with the high sequence similarities between ϕO104 and ϕPA8 (85% query coverage, 98% identity; Additional file 3: Figure S2).

### Overview of the genes found upregulated in the Stx2a phage lysogens in comparison to naive MG1655

Further analysis using the STRING database of protein-protein interactions [34] revealed that, among others, sulfur-related genes, as well as genes involved in the SOS response, motility and chemotaxis were upregulated in the Stx2a phage lysogens in comparison to naive MG1655 (Additional file 3: Figure S3). Several genes, which were previously described to be responsive to sulfur availability [35], i.e. *yeeE* encoding an inner membrane protein, *yeeD* coding for a putative sulfur transferase and *ydjN* encoding a cystine/cysteine/sulfocysteine: cation symporter were among the most strongly upregulated genes (6–20 fold) in both lysogens. In addition, the genes involved in the sulfur metabolism *cysD*, *cysJ*, *cysK* and *cysPUW* were significantly upregulated in MG1655::ϕPA8, whereas in MG1655::ϕO104 these genes were also detected upregulated but below the padj < 0.1 applied in our analysis (Additional file 1, Additional file 2 and Additional file 3: Table S2).

The most abundant class of upregulated genes in both MG1655::ϕO104 and MG1655::ϕPA8 was those that form the SOS response-regulon. These genes included *recN*, which codes for the DNA repair protein RecN, *recA*, which codes for the DNA recombination/repair protein RecA, and *umuDC,* encoding the DNA polymerase V subunits, among others (Additional file 3: Figure S3). A detailed investigation into the SOS response-related upregulated genes detected in the Stx2a phage lysogens and their biological relevance will be presented in a separate publication (manuscript in preparation).

In addition, genes involved in motility and chemotaxis were found upregulated in both lysogens, i.e. *fliC* coding for flagellin, *cheW* and *tar* coding for the chemotaxis proteins CheW and Tar, respectively (Additional file 3: Figure S3). It has been previously reported that MG1655 lysogenized with the Stx2a phage ϕMin27 displays elevated levels of flagellar gene transcripts and has enhanced swimming motility [29]. Similarly, we found here that MG1655::ϕO104 expressed 1.8 fold higher levels FliC and displayed a significantly increased swimming motility compared to parental naive MG1655 strain (Additional file 3: Figure S4).

Several metabolic genes were also found upregulated in both Stx2a phage lysogens. These included the genes involved in mixed acid fermentation *ackA* encoding the acetate kinase, *ldhA* coding for the D-lactate dehydrogenase and *aceE* coding for the pyruvate dehydrogenase complex E1 component (also functioning in linking glycolysis to the TCA cycle), as well as *ndh* coding for the NADH dehydrogenase II.

### Overview of the genes found downregulated in the Stx2a phage lysogens in comparison to naive MG1655

**Fig. 2:**
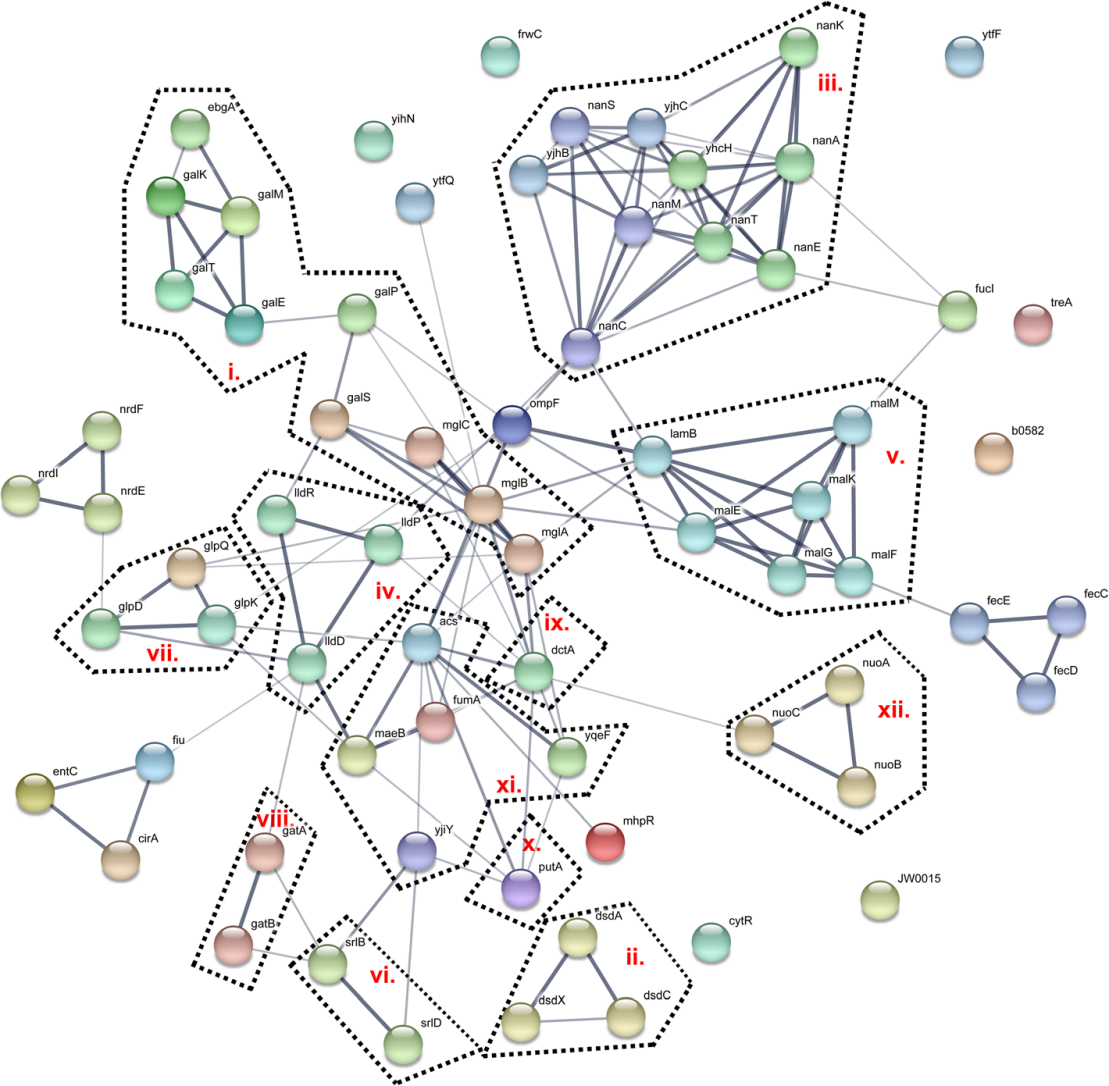
Interaction network of the genes found downregulated in MG1655::ϕO104. The list of genes found downregulated (*n* = 69) in MG1655::ϕO104 in comparison to naive MG1655 was analyzed using the STRING database of protein-protein interactions. The network summarizes the predicted associations for the proteins encoded by the upregulates genes detected in our analysis. The network nodes are the proteins and the edges represent the predicted functional associations. The thickness of the line indicates the degree of confidence prediction of the interaction. The genes encode proteins involved in the assimilation of: (i) galactose; (ii.) D-serine; (iii.) sialic acid; (iv.) L-lactate; (v) maltose; (vi.) sorbitol; (vii.) glycerol; as well as (viii.) components of the galactitol-specific PTS enzyme II; (ix.) C4-dicarboxylate transporter; (x.) dehydrogenase of the proline degradation pathway; (xi.) proteins of the pyruvate metabolism; and (xii.) subunits of the NADH dehydrogenase I

Interestingly, our STRING analysis revealed that the majority of the downregulated genes in the Stx2a phage lysogens were involved in carbon source transport and metabolism (Fig. 2 and Additional file 3: Figure S5). Both MG1655::ϕO104 and MG1655::ϕPA8 were characterized by reduced expression of genes involved in the assimilation of: (i) galactose, e.g. *mglBAC* operon coding for subunits of the D-galactose ABC transporter (transports also glucose) and *galETKM* operon encoding enzymes involved in the galactose degradation I (Leloir) pathway; (ii) D-serine, e.g. *dsdXA* operon coding for the D-serine transporter and ammonia-lyase; (iii) sialic acid, e.g. *nanS* encoding the N-acetyl-9-O-acetylneuraminate esterase and the sialic acid catabolic operon *nanATEK*-*yhcH*; (iv) L-lactate, i.e. the *lldPRD* operon involved in L-lactate transport and degradation; (v) maltose, i.e. *malK*-*lamB*-*malM* operon encoding the maltose outer membrane channel and phage lambda receptor protein LamB and the *malEFG* operon encoding subunits of the maltose ABC transporter (transports also glucose); and (vi) sorbitol, i.e. *srlD* coding for the sorbitol-6-phosphate 2-dehydrogenase and *srlB* for component of the sorbitol-specific PTS enzyme IIA. In addition, genes involved in the utilization of (vii) glycerol, e.g. *glpQ* coding for the glycerophosphoryl diester phosphodiesterase and *glpK* encoding the glycerol kinase were found downregulated in MG1655::ϕO104, whereas *glpQ* together with *dhaKLM* operon coding for the dihydroxyacetone kinase (glycerol degradation V) in MG1655::ϕPA8. The *gatABC* operon encoding components of the (viii) galactitol-specific PTS enzyme II was turned down in the MG1655::ϕO104 lysogen and the genes involved in the utilization of ribose, i.e. *rbsD*, *rbsB*, *rbsK* and fructuronate/ glucuronate, i.e. *uxuAB* and *gntP* were found significantly downregulated only in the MG1655::ϕPA8 lysogen. Furthermore, both Stx2a phage lysogens downregulated *dctA* coding for (ix) C4-dicarboxylate transporter, responsible for the uptake of fumarate, succinate, L-aspartate etc. under aerobic conditions and *putA* involved in the (x) proline degradation pathway. Last but not least, MG1655::ϕO104 and MG1655::ϕPA8 bore downregulated genes involved in the metabolism of (xi) pyruvate, e.g. *yjiY* encoding a pyruvate:H^+^ symporter, *fumA* encoding fumarase A, *acs* encoding acetyl-CoA synthetase, *maeB* coding for malate dehydrogenase, and *nuoABC* coding for the (xii) NADH dehydrogenase I (*nuoAB* is found significant only in MG1655::ϕO104; Fig. 2 and Additional file 3: Figure S5).

### Stx2a phage lysogens display moderate to severe growth defects in minimal medium supplemented with single carbon sources

Given the changes in gene expression, we next determined whether the downregulation of the genes involved in carbon transport and utilization in the Stx2a phage lysogens affected the bacterial phenotype. As a first step, we compared the growth of MG1655::ϕO104 and MG1655::ϕPA8 in minimal medium supplemented with single carbon sources, i.e. glucose, maltose, L-Lactate, galactose, N-acetylneuraminic acid (sialic acid) and ribose to that of naive MG1655. We found that, as compared to the parental MG1655 strain, the Stx2a phage lysogens exhibited significant differences in the generation time and/or final OD_595_ after 24 h of growth with the tested compounds (Table 1, Additional file 3: Figure S6). The Stx2a phage lysogens displayed moderate growth defects in glucose, maltose and sialic acid (reaching up to 46–86% of the MG1655 final OD_595_) and more severe ones in ribose, galactose and lactate (reaching only 7–43% of the MG1655 final OD_595_). We detected no significant difference in the bacterial cell size of the three strains after growth with glucose, galactose and ribose, suggesting that the differences in the OD_595_ measurements reflected real differences in the cell number in the cultures (Additional file 3: Figure S7). Interestingly, the observed growth defects were independent of the type of starter culture used, i.e. culture grown to exponential phase (the condition used in RNA-seq analysis) or overnight culture (Table 1).

**Table 1:** Growth characteristics of MG1655 and the Stx2 lysogens in minimal medium supplemented with singe carbon sources

Strain	Growth medium^a^	OD = 0.4 pre-culture		ON pre-culture	
		Generation time [min]^b^	Final OD_595_^b^	Generation time [min]^b^	Final OD_595_^b^
MG1655	MM glucose	88 ± 3	0.41 ± 0.00	81 ± 2	0.39 ± 0.01
MG::ϕO104		103 ± 1***	0.31 ± 0.01***	106 ± 3***	0.33 ± 0.02**
MG::ϕPA8		106 ± 2***	0.35 ± 0.00***	101 ± 6**	0.34 ± 0.01**
MG1655	MM maltose	120 ± 3	0.34 ± 0.00	111 ± 1	0.32 ± 0.00
MG::ϕO104		187 ± 13***	0.23 ± 0.01***	184 ± 5***	0.15 ± 0.00***
MG::ϕPA8		206 ± 4***	0.24 ± 0.00***	200 ± 9***	0.18 ± 0.01***
MG1655	MM L-lactate	175 ± 1	0.25 ± 0.02	170 ± 1	0.29 ± 0.01
MG::ϕO104		295 ± 43**	0.10 ± 0.01***	949 ± 20***	0.05 ± 0.00***
MG::ϕPA8		218 ± 7	0.09 ± 0.00***	526 ± 19***	0.05 ± 0.01***
MG1655	MM galactose	172 ± 5	0.38 ± 0.00	166 ± 2	0.35 ± 0.00
MG::ϕO104		193 ± 12*	0.16 ± 0.00***	254 ± 10**	0.09 ± 0.00***
MG::ϕPA8		224 ± 1**	0.10 ± 0.01***	319 ± 27***	0.07 ± 0.00***
MG1655	MM sialic acid	102 ± 2	0.44 ± 0.00	91 ± 0	0.43 ± 0.00
MG::ϕO104		108 ± 1**	0.30 ± 0.00**	109 ± 2***	0.32 ± 0.00***
MG::ϕPA8		105 ± 1	0.30 ± 0.00**	114 ± 3***	0.33 ± 0.00***
MG1655	MM ribose	180 ± 4	0.35 ± 0.00	177 ± 4	0.29 ± 0.00
MG::ϕO104		456 ± 9***	0.03 ± 0.00***	457 ± 46***	0.02 ± 0.00***
MG::ϕPA8		791 ± 61***	0.02 ± 0.01***	583 ± 29***	0.02 ± 0.00***
MG1655	LB	32 ± 1	0.97 ± 0.02	33 ± 1	0.94 ± 0.00
MG::ϕO104		33 ± 0	0.12 ± 0.01***	33 ± 1	0.12 ± 0.01***
MG::ϕPA8		32 ± 1	0.28 ± 0.02***	34 ± 1	0.27 ± 0.00***

*The strains displaying significantly different growth characteristics in comparison to MG1655 are marked: * *p* < 0.05, ** *p* < 0.01, *** *p* < 0.001

^a^Cells were grown either in minimal medium (MM) supplemented with 0.2% of single carbon source or in the nutrient rich LB medium

^b^The values represent mean and standard error of the mean of three biological replicates

We detected no significant difference in the doubling time among the strains in the nutrient rich LB medium used here as control (Table 1). However, both Stx2a phage lysogens also reached a significantly lower final OD_595_in LB than did naive MG1655. We noted that the growth curves of the Stx2a phage lysogens grown in LB displayed a drop in the OD_595_ shortly after the onset of stationary phase suggesting this transition triggered cell death (Additional file 3: Figure S6). We hypothesized that prophage induction may be responsible for this increase in cell mortality. Consistent with this idea, quantitative PCR analysis revealed an extremely elevated *stx2a* copy number in MG1655::ϕO104 after an overnight incubation in LB in comparison to the exponential starter culture (ca. 7.8 × 10^3 fold increase; Additional file 3: Table S4). In contrast, the *stx2a* levels in MG1655::ϕO104 grown in minimal medium either did not increase (with glucose) or showed only ca. 30–60 fold elevation (with the rest of the tested carbon courses). Moreover, there was no correlation between the fold increase in the *stx2a* copies and the degree of MG1655::ϕO104 growth retardation in the corresponding medium. These observations suggested that the detected growth defects of the Stx2a phage lysogens in minimal medium supplemented with the above listed carbon sources could not be attributed to increase in the phage induction frequency.

### Phenotype microarrays revealed additional differences in the ability of Stx2a phage lysogens to utilize substrates as a sole source of carbon

**Fig. 3:**
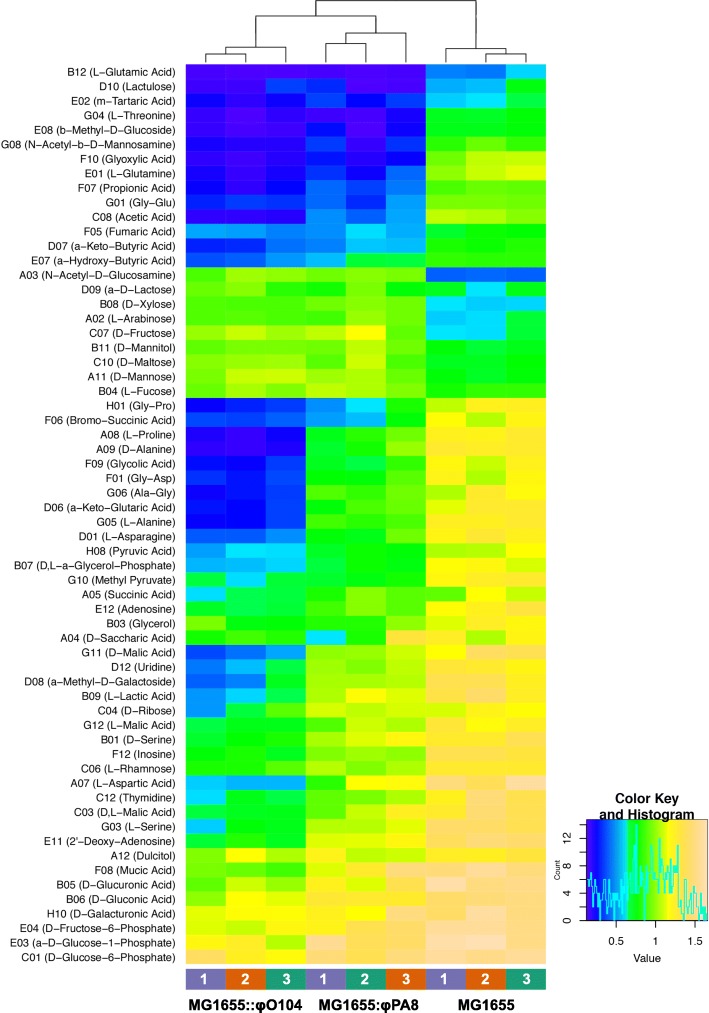
Heatmap of the respiration potential of naive MG1655 and the Stx2a phage lysogens analyzed with the BIOLOG PM1 plate. The heatmap shows a subset of substrates, with which the Stx2a phage lysogens display significantly reduced or increased respiration in comparison to naive MG1655. Three biological replicates per strain were analyzed. The degree of respiration with each substrate is color coded based on the provided color key. The tree on top of the map shows the relationship between the analyzed samples based on their overall respiration pattern

We used the BIOLOG phenotype microarray system and the plate PM1 MicroPlate™ Carbon Sources to further explore the effect of the observed prophage-dependent changes in gene expression on the growth/respiration phenotypes of MG1655::ϕO104, MG1655::ϕPA8 and naive MG1655 (Fig. 3 and Additional file 3: Figure S8 and Table S5). The BIOLOG PM1 profile obtained with the MG1655 strain was in good agreement with the results from the five independent datasets summarized in the EcoCyc *E. coli* Database [36]. In our hands, MG1655 showed no or poor respiration with 23 of the 95 tested substrates, while it was able to successfully utilize with different efficiency the remaining 72 substrates (Additional file 3: Figure S8). Both Stx2a phage lysogens exhibited respiration profile significantly different from that of the naive MG1655. In total, 61 and 55 substrates were differentially assimilated by MG1655::ϕO104 and MG1655::ϕPA8, respectively (Additional file 3: Table S5). In the majority of cases, 53/61 in MG1655::ϕO104 and 47/55 in MG1655::ϕPA8, the Stx2a phage lysogens had a significantly reduced respiration in comparison to naive MG1655 (Fig. 3 and Additional file 3: Figure S8). With regard to our RNA-seq results, the BIOLOG analysis confirmed the reduced respiration of the Stx2a phage lysogens with the substrates D-serine (significant only in MG1655::ϕO104), L-lactic acid, glycerol, ribose (significant only in MG1655::ϕO104), proline, methyl pyruvate, etc. Both MG1655::ϕO104 and MG1655::ϕPA8 displayed reduced respiration with sorbitol as well, however below the level of significance applied in our analysis. Even though MG1655 displayed initially higher respiration rate with galactose, glucose and maltose, the Stx2a phage lysogens were able to reach the respiration potential of MG1655 and, in the case with maltose, to significantly surpass it (Additional file 3: Figure S8 and Table S5). Interestingly, the MG1655::ϕO104 and MG1655::ϕPA8 displayed markedly reduced respiration with the majority of gluconeogenic and TCA substrates tested, e.g. amino acids (proline, glutamine, threonine, etc.), nucleosides (thymidine, uridine, adenosine, etc.), carboxylic acids (acetic acid, propionic acid, glyoxylic acid, etc.) and dicarboxylic acids (succinic acid, malic acid, mucic acid, etc.). Of the Stx2a phage lysogens, MG1655::ϕO104 exhibited the most drastic growth/respiration defects, assimilating 30 substrates less efficiently than even MG1655::ϕPA8 (Additional file 3: Table S5). Both the Stx2a phage lysogens displayed higher respiration with L-arabinose, N-acetyl-D-glucosamine, mannose, L-fucose (significant only in MG1655::ϕPA8), xylose, mannitol, fructose, maltose, α-D-lactose (significant only in MG1655::ϕO104) than did naive MG1655 (Fig. 3 and Additional file 3: Figure S8 and Table S5).

### Phage encoded factor(s) apart from CI and Cro are responsible for the carbon utilization phenotypes of Stx2a phage lysogens

The data presented in this study were obtained with two independent Stx2a phage lysogens with distinct integration sites [4, 33], indicating that phage encoded factor(s) rather than mutations in the bacterial chromosomal backbone mediate the changes in carbon utilization of MG1655::ϕO104 and MG1655::ϕPA8 in comparison to naive MG1655. Moreover, since the majority of the changes in the transcriptome and phenotype were detected in both lysogens, the factor(s) responsible for them should be shared between these phages. Besides their normal roles in regulating phage transcription, the CII and Cro proteins in other Stx2a-encoding phages have already been shown to regulate the expression of some bacterial host genes [30–32]. The ϕO104 and ϕPA8 apparently do not encode a CII protein and express only the CI repressor and the Cro anti-repressor [33]. The sequences of these two proteins are 100% identical between the two phages. Our transcriptome data showed that these regulators were expressed on the RNA level, with *cI* being the one more actively transcribed in both lysogens (Additional file 3: Table S6), a feature that was in agreement with its function in maintaining lysogeny [37]. In order to determine whether these phage regulators contribute to the observed changes in carbon source utilization, we cloned *cI* and *cro* genes into the low copy pWKS30 vector under the control of their native promoters and validated their expression on the mRNA level (see Methods and Additional file 3: Table S7). We found that MG1655 bearing either pWKS30-*cI* or pWKS30-*cro* exhibited no significant differences in the doubling time and final OD_595_ detected in growth experiments with minimal medium supplemented with 0.2% glucose, maltose, L-lactate, galactose, glycerol and ribose in comparison to cells with empty vector (Additional file 3: Figure S9). As measured using the BIOLOG PM1 plate, we found that the strains carrying these plasmids exhibited virtually identical respiration profile as did naive MG1655 (Additional file 3: Figure S10). These results indicated that phage encoded factor(s) apart from CI and Cro are responsible for the carbon utilization phenotypes detected in MG1655::ϕO104 and MG1655::ϕPA8.

## Discussion

Here, we analyzed the effects of the carriage of two closely sequence related Stx2a phages: ϕO104 from the exceptionally pathogenic 2011 *E. coli* O104:H4 outbreak strain and ϕPA8 from an *E. coli* O157:H7 isolate on *E. coli* K-12 host. Both quantitative RNA-seq analysis and phenotypic investigations revealed a profound impact of the Stx phage carriage on MG1655 carbon source utilization.

The strong correlation between our transcriptome and phenotype analyses indicates that the majority of detected changes in gene expression in the Stx2a phage lysogens are robust as they apparently result in stable phenotypic changes. The only major exception to this correlation is the observed lack of significant reduction in the respiration detected with sorbitol, galactose, glucose and maltose in the BIOLOG assay. However, the finding that growth in these carbon sources is accompanied by initial growth retardation of the lysogens in comparison to naive MG1655 suggests that in this case the prophage-dependent alteration in host metabolism occurs only transiently, i.e., it is a growth phase-specific phenotype. The discrepancy between the results of the BIOLOG microarrays with glucose, maltose and galactose and that of the minimal medium experiments with these carbon sources suggests that the growth retardation/reduced respiration phenotypes are more pronounced after a shift from nutrient rich to poor medium (Additional file 3: Figures S6 and S8).

We wished to gain insight into how Stx prophage carriage affects *E. coli* host cell transcription. To this end, we compared our RNA-seq results with the previously published high throughput data sets obtained from *E. coli* host lysogens bearing the Stx2a phages ϕMin27 or ϕ24_B_ [29, 30]. We find that there is limited to no correlation between the transcriptional effects of ϕO104 or ϕPA8 with cells bearing either of these two Stx2a phages. We suggest that the sequence heterogeneity of these Stx2a phages plays the major role in determining the differences in the transcriptome between these individual strains. Notably, ϕMin27 and ϕ24_B_ share only limited sequence similarity to the Stx2a phages used in our analysis (59–64% query coverage, 97–98% identity). However, differences in the methodologies used and/or experimental set up may also contribute to the discrepancies. For example, the study on the ϕMin27 impact on host transcription is based on microarray gene expression data [29]. Similarly, Veses-Garcia et al. applied next generation sequencing in combination with an SOS-triggered phage induction in order to select for the ϕ24_B_ lysogeny-specific transcriptional response. They excluded all differentially regulated genes detected upon norfloxacin treatment (phage induction) from their analysis [30]. This approach may mask stable changes in the host gene expression, which might be present both under phage lysogeny and in the recovery phase upon the norfloxacin treatment, repressing expression of genes involved in aerobic metabolism, while stimulating those involved in anaerobic respiration.

Our RNA-seq data show that carriage of the Stx2a phages ϕO104 or ϕPA8 results in a reprogramming of the host cell’s central metabolism (Fig. 2, Additional file 3: Figures S3 and S5 and Tables S2 and S3). This reprogramming might be aimed at allowing *E. coli* to grow and compete better under anaerobic conditions. On one side Stx2a prophage carriage represses (directly or indirectly) the expression of several genes involved in aerobic carbon metabolism. These include genes that encode the TCA enzymes fumarase A (*fumA*) and malate dehydrogenase (*maeB*) and subunits of the NADH dehydrogenase I (i.e. *nuoABC*), which is part of the oxidative phosphorylation pathway. On the other hand, Stx2a phage carriage activates genes encoding enzymes involved in fermentative/anaerobic carbon metabolism (i.e., *ldhA*, *ace* and *ackA*) and NADH dehydrogenase II (i.e., *ndh*), which in comparison to NADH dehydrogenase I recycles NADH without generating an electrochemical gradient and with less efficiency [38].

Significantly, when accompanied by upregulation of *ndh*, the downregulation of the *nuo* genes would promote assembly of a respiratory chain that is not optimal for the efficient oxidation of NADH to NAD^+^. This change should lead to NADH accumulation, which in turn would reduce TCA cycle enzyme activity via the allosteric inhibition of malate dehydrogenase [39] and citrate synthase [40] (enzymes shared between TCA and glyoxylate cycle). Moreover, *nuo* mutants, which grow poorly on acetate as a single carbon source, are also known to be unable to efficiently grow on amino acids, whose metabolism is TCA cycle-dependent. This growth defect presumably results from suppression of TCA cycle activity due to high NADH/NAD^+^ [41].

Our results support this idea, i.e., we find that the Stx2a phage lysogens have a decreased ability to utilize TCA cycle and gluconeogenic substrates, e.g. amino acids, carboxylic and dicarboxylic acids (Fig. 3 and Additional file 3: Figure S8). This increase in the availability of intracellular NADH in the Stx2a phage lysogens should also lead to a shift to fermentative/anaerobic carbon metabolism. Indeed, it was reported that artificially increasing the availability of NADH during aerobic growth induces the production of fermentation end products (e.g. ethanol, lactate) [42].Consistent with this prediction, we find that expression of *ldhA*, *ace* and *ackA* is upregulated in the Stx2a phage lysogens.

**Fig. 4:**
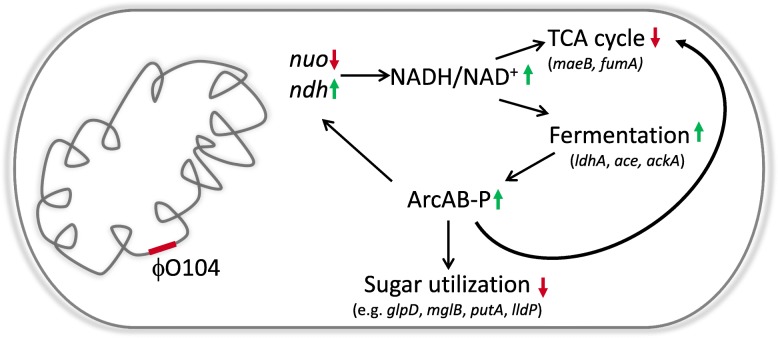
Stx2a phage-dependent changes geared at reprogramming MG1655 metabolism. A schematic representation of the proposed model for the Stx2a phage-dependent changes in the gene expression, NADH/NAD^+^ ratio and level of ArcAB phosphorylation leading to profound differences in the carbon source utilization. For more details refer to the text in the discussion. Legend: red arrow pointing down = downregulation/decrease; green arrow pointing up = upregulation/increase, ArcAB-P = phosphorylated ArcAB

Fermentation products and elevated NADH/NAD^+^ ratios are linked to the level of phosphorylated and active form of the two component system ArcAB [43–45], which is one of the global regulators coordinating gene expression to changes in O_2_ availability [46]. Therefore, it comes as no surprise that there are multiple overlaps between the Stx2a phage-carriage-dependent downregulated genes detected here and the ArcA regulon [47]: *nuo*, the TCA cycle genes *fumA* and *maeB*, genes involved in carbon source transport and metabolism *glpD*, *mglB*, *putA*, *lldP* and others. The above discussed Stx phage-dependent changes in the gene expression geared at reprogramming *E. coli* K-12 metabolism are summarized in Fig. 4. It should be nevertheless noted that our model explains the majority but not all the changes detected in our analysis.

The ability of both commensal and pathogenic *E. coli* to colonize the intestine is governed by the relative efficiencies with which a given strain can use a particular carbon source, which is, in turn, determined by the activities of a cell’s central metabolic pathways [48]. For example to efficiently colonize streptomycin-treated mice, MG1655 relies on its ability to metabolize N-acetylneuraminic acid, N-acetyl-D-glucosamine, arabinose, gluconate and fucose [49]. Our results show that carriage of either ϕO104 or ϕPA8 substantially decrease MG1655’s ability to grow on N-acetylneuraminic acid and gluconate while increasing its ability to utilize the other sugars (Table 1 and Fig. 3). If the results of our in vitro studies accurately predict what sugars a cell can utilize in vivo, the Stx2a phage lysogens should outcompete naive MG1655 in niches with available arabinose, fucose and N-acetyl-D-glucosamine, while be readily eliminated in the ones with N-acetylneuraminic acid and gluconate. Interestingly, certain EHEC strains carry additional functional *nanS*-homologues alleles, which are prophage-encoded [50–52]. In respect to our data, multiple *nanS* copies would be beneficial to compensate for potential Stx phage-dependent decrease in the ability to utilize N-acetylneuraminic acid due to downregulation of the native *nan* operons.

Even though *E. coli* grows best on sugars (mono- and disaccharides), it can also grow on amino acids and other substrates for the TCA cycle [48]. However, TCA cycle and gluconeogenic mutants exhibit no defects in colonization of the streptomycin-treated mouse intestine and thus exclude a major role of amino acids catabolism in colonization of MG1655 [53]. In addition, MG1655 use glycolytic but not gluconeogenic substrates for growth in this mouse model when fed alone or together with *E.coli* O157:H7 [54]. However, both aerobic and anaerobic respiration mutants of MG1655 and *E. coli* O157:H7 are eliminated by competition with wild type strains and respiration of oxygen provides greater colonization advantage than anaerobic respiration in the streptomycin-treated mouse intestines to both *E. coli* species [55, 56]. Therefore, it remains unclear if the decreased potential of the Stx2a phage lysogens to utilize amino acids, dicarboxylic acids and other gluconeogenic substrates detected here under aerobic conditions (Fig. 3) would compromise their colonization efficiency in comparison to naive MG1655.

The downregulation of sugar import and catabolism, as well as of aerobic metabolism (TCA cycle and oxidative phosphorylation) might in addition contribute to the survival of the Stx2a phage lysogen population, e.g. by stimulating persister cell formation [57, 58]. Persister cells are genetically unaltered subpopulations of cells that are locked into “dormant, non-dividing phase” and can thus survive otherwise lethal doses of antibiotics [59]. Intriguingly, among the upregulated genes in both MG1655::ϕO104 and MG1655::ϕPA8 we identified genes, which have previously been described to be involved in the formation of persister cells, i.e. *tisB*, encoding the toxin peptide of Type I toxin-antitoxin system [60] and *ldhA* [61]. The maintenance of an increased number of persisters in otherwise favorable growth conditions may be especially important for Stx phage lysogens, as a stress depended induction of the phage might otherwise result in a complete collapse of the bacterial population. As other potential susceptible hosts would experience the same stress in such an environment, stable lysogenization of a new host would be impossible and thus eventually endanger the long-term survival of the phage itself.

Our heterologous expression experiments in MG1655 revealed that phage encoded factor(s) apart from CI and Cro are responsible for the carbon utilization phenotypes detected in MG1655::ϕO104 and MG1655::ϕPA8. Experiments involving systematic heterologous expression of phage regions in MG1655 or phage truncation experiments in the Stx2a phage lysogens will be necessary to discover the so far unknown regulatory determinant(s). Determining these factor(s) will facilitate the evaluation of the biological meaning of the here described Stx2a phage-dependent effects on the bacterial host and in particular if these traits are shared with other phages or they are rather Stx2a phage-, or even ϕO104- and ϕPA8-specific effects.

## Conclusion

Here, we use quantitative RNA-seq to demonstrate that Stx2a phage carriage has a previously unforeseen strong impact on *E. coli* K-12 host gene expression governing carbon source transport and metabolism. Subsequent phenotypic tests including microarrays show that the lysogeny is indeed associated with the according changes in the carbon source utilization. Thus, our work confirms previous observations that Stx phage carriage not only provides the bacterial host with the ability to produce Stx, but that it can also have an impact on its gene expression and phenotype. The strong correlation between our transcriptome and phenotype analyses indicate that the majority of detected changes in the gene expression patterns of the analyzed Stx2a phage lysogens are robust as they result in stable phenotypic changes. The Stx2a prophage appears to reprogram the carbon metabolism of its bacterial host by turning down aerobic metabolism in favour of mixed acid fermentation. This results in the decreased ability of Stx2a phage lysogens to utilize TCA cycle and gluconeogenic substrates, e.g. amino acids, carboxylic and dicarboxylic acids, and other carbon sources. In addition, the Stx2a phage carriage apparently leads to an enhanced respiration with several sugars that comprise part of the intestinal mucus.

To our knowledge, this is the first study that describes that the Stx phage carriage results in profound changes in metabolism and carbon source utilization potential of the host and that these changes are very likely based on Stx phage-dependent reprogramming of the host cell’s transcriptional profile. The phage factor(s) that are responsible for these dramatic differences in the host’s transcriptome and phenotype are, as yet, unidentified.

## Methods

### Strains

The *E. coli* K-12 strain MG1655 [62] was used as the recipient for the ϕO104 and ϕPA8 Stx2a phages. Phages ϕO104 and ϕPA8 were isolated from the parental EHEC strains by treating exponential phase cells grown in LB plus 10 mM MgSO_4_ with 5 μg/mL mitomycin C. After an additional 4 h of growth at 37 °C to allow for phage growth and cell lysis, an aliquot of the lysate was extracted with CHCl_3_ and the cellular debris pelleted by centrifugation at 13,000 *g*. Dilutions of the phage-containing supernatants were spotted on a lawn of MG1655 growing in soft agar overlaid on an LB agar plate and the plates incubated overnight at 37 °C to allow the formation of phage plaques. Putative lysogens were picked from the center of these plaques and streaked on an LB agar plate. The successful lysogenization was verified by colony PCR with primers PB_1 & PB_2 binding within *stx2a* (Additional file 3: Table S8). The insertion of ϕO104 in *wrbA* of MG1655 (MG1655:: ϕO104) was confirmed by PCR with primers PB_4 & PB_5 and of ϕPA8 in *argW* (MG1655:: ϕPA8) with PB_7 & PB_8.

### Bacterial growth and RNA preparation for transcriptome analysis

The bacterial growth and RNA preparation for the RNA-seq experiment were performed as previously described [63] with the following modifications. The experiments were performed with 3 biological replicates per strain. Overnight cultures were diluted till OD_600_ of 0.005 in LB medium (10 g/L tryptone, 5 g/L yeast extract, 5 g/L NaCl) and cells were grown at 37 °C, 180 rpm to an OD_600_ of 0.4–0.6. After the treatment with stop solution (5% phenol/95% ethanol), cells pellets were immediately lysed with cell lysis buffer (85 mg/mL lysozyme in TE buffer, pH 8). One mL of Trizol reagent was added and samples were stored at -20 °C till the RNA isolation protocol was resumed.

### RNA-seq: cDNA library construction, sequencing and data analysis

RNA preparations were treated with RiboZero (Illumina) and converted to cDNA libraries using NEBNext Ultra Directional RNA Library Kit following the manufacturers’ instructions. cDNA libraries were sequenced on NextSeq 550 System using NextSeq 500/550 Mid-Output kit (Illumina). The quality of the raw sequenced reads was checked using FastQC v.0.11.2 [64]. Adapters were removed using cutadapt v.1.14.1 [65] and reads were reverse complemented with FASTX v.0.0.14 [66]. Data was mapped to the reference genomes of *E. coli* MG1655 (U00096.3), ϕPA8 (KP682374.1) and ϕO104 (3,256,115 to 3,317,011 from NC_018658.1) using READemption v.0.4.3 [67] and segemehl v.0.2.0 [68]. Differential gene expression analysis was performed with READemption and DESeq2 v.1.16.1 package in R [69]. Changes in the gene expression ≥1.5 fold (log2fold ≥ 0.58) with adjusted *p* value for multiple comparison (padj) < 0.1 were considered significant.

### Growth kinetics, cell size, Stx2a phage presence and *stx2a* gene copy measurements in minimal medium supplemented with single carbon sources

Overnight or exponential (OD_600_ = 0.4–0.6) starter LB cultures were diluted 1:200 in N-C- minimal medium [70] with 10 mM ammonium chloride as the nitrogen source and 0.2% of single carbon source. The following carbon sources were used: glucose (Sigma, # G7528), maltose monohydrate (Sigma, # M5895), sodium L-lactate (Sigma, #71718), galactose (Sigma, #G0750), N-acetylneuraminic acid (Sigma, #A2388) and ribose (Sigma, # R7500). The growth kinetic measurements over 24 h were performed as previously described [71]. Due to the use of LB starter cultures the strains were characterized by diauxic growth curves in minimal medium. The minimal doubling time with each carbon source was calculated using the linear region of the second phase of the growth curve.

After the 24 h incubation, the samples were further analyzed by measuring the cell size of the strains, verifying the presence of the Stx2a phage and determining the *stx2a* copy number in the lysogens. The cell size of the strains was controlled by light microscopy. Briefly, 100 μL of the samples grown in minimal medium with glucose, galactose and ribose were mixed with 800 μL PBS and 100 μL 37% formaldehyde. After 1 h incubation at room temperature, 5 μL were spotted on a slide and visualized using Nikon Eclipse Ci microscope and 40x magnification. The cell size was determined with the NIS-Elements D 4.60.00 imaging software. The presence of the Stx2a phage in the lysogens was routinely controlled by diluting the samples to 10^6 or 10^7 and plating, followed by colony PCR with *stx2a* primers (PB_1 and PB_2) to screen 20 randomly selected colonies per growth condition. To determine semi-quantitatively the *stx2a* copy numbers in MG1655::ϕO104 after the growth in minimal medium, 50 μL were boiled for 10 min and used as template for quantitative PCR (Bio-Rad) with *stx2a* (PB_1 & PB_2) and *gapA* (PB_9 & PB10) primers (Additional file 3: Table S8). The linearity of the primers was tested by 10 fold serial dilutions of gDNA template from *E. coli* O104:H4. Data was analyzed using the Bio-Rad CFX Manager 3.1. The increase in the *stx2a* copies was calculated using the ΔΔCt method with *gapA* being the control gene and the starter culture (exponential LB culture) set as control sample.

### Genome comparisons of Stx2a phages

The phages ϕO104 and ϕPA8 were aligned using Mauve with default parameters. The similarities (% query coverage, % identity) between the phage genomes were analyzed using NCBI blastn [72]. The phages ϕ24B (HM208303.1) and ϕMin27 (EU311208) were additionally included in the blastn analysis.

### BIOLOG phenotype microarrays: workflow and data analysis

The BIOLOG phenotype microarray experiments with PM1 carbon sources were performed following the manufacturer’s instruction with the following modification: the glycerol stocks of the strains were plated on Columbia Blood Agar (Oxoid). The growth kinetics measurements of the PM1 plates were done in a TECAN Infinite F200 instrument by determining the OD_595_ every 15 min for 24 h at 37 °C without shaking. BIOLOG data was analyzed and visualized using the opm package v.1.3.77 for R [73]. Briefly, after importing of kinetic raw data and metadata integration, descriptive curve parameters (lag phase (λ), respiration rate (μ), maximum curve height (A) and Area Under the Curve (AUC)) were estimated using do_aggr function via spline-fitting. Statistical analysis of growth curves of the three strains (naive MG1655, MG1655::ϕO104 and MG1655::ϕPA8) in three independent repetitions was performed with opm_mcp method, which internally accounts for multiple comparisons.

### Heterologous expression of Cro and CI in MG1655

The Stx2a phage regions from position − 105 to + 51 relative to the annotated *cro* ORF and from position − 88 to + 90 relative to the annotated *cI* ORF (encompassing their predicted promoter regions) were amplified from total DNA from MG1655::ϕO104 using the Phusion Polymerase (Thermo Scientific) and the primers PB_11 & 12 and PB_13 & 14 (Additional file 3: Table S8), respectively. The fragments were ligated into the low copy number plasmid pWKS30 [74] linearized with the restriction enzyme SmaI and transformed in chemically competent NEB 5-alpha *E. coli* cells (NEB). Plasmids carrying the constructs in the same orientation (cloned ORFs downstream of the *lac* promoter in pWKS30) were selected by colony PCR using primers M13_rev & PB_12 or PB_13. Sanger sequencing was used to verify the correct sequence of the cloned constructs. The plasmids pWKS-*cI* and pWKS-*cro* were then transformed in electrocompetent MG1655 cells. The expression of *cI* and *cro* in MG1655 pWKS-*cI* and MG1655 pWKS-*cro*, respectively, was verified by reverse transcription polymerase chain reaction (RT-PCR) in total RNA isolated form cells grown to exponential phase in LB or minimal medium supplemented with 0.2% glucose. RT-PCR was performed with OneStep RT-PCR kit (Qiagen) and the primers PB_15&16 (*cI*) and PB_17&18 (*cro*).The relative normalized expression of *cI* and *cro* was calculated using the ΔΔCt method with *gapA* being the control gene (quantified with primers PB_9&PB_10) and MG1655::ϕO104 set as a control sample.

### Statistical analysis and data visualization

Statistical analysis of data other than RNA-seq and BIOLOG datasets was done using R v.3.4.0 computing environment [75]. Welch two sample t-test was used to compare the means of two groups of samples. ANOVA was used to assess the difference between more than two sample groups. Pairwise comparisons between groups were performed using t-tests with corrections for multiple testing using the Holm method. Figures were created using opm and ggplot2 [76] packages in R and Adobe Photoshop CC 2017.

## Additional files

Additional file 1: DESeq2 results using MG1655 as the reference librabry (divisor) and MG1655::φO104 as the comparison library (numerator). (XLSX 633 kb)

Additional file 2: DESeq2 results using MG1655 as the reference librabry (divisor) and MG1655::φPA8 as the comparison library (numerator). (XLSX 697 kb)

Additional file 3: **Table S1.** Overview of sequenced and mapped reads. **Figure S1.** Principal component analysis (PCA). **Table S2.** Up- and downregulated genes in MG1655::ϕO104 in comparison to naive MG1655. **Table S3.** Up- and downregulated genes in MG1655::ϕPA8 in comparison to naive MG1655. A. Upregulated genes. B. Downregulated genes. **Figure S2.** Genome alignment of ϕO104 and ϕPA8. **Figure S3.** Interaction analysis of the genes found upregulated in the Stx2 lysogens. **Figure S4.** Stx2 phage carriage enhances FliC expression and motility. **Figure S5.** Interaction analysis of the genes found downregulated in MG1655::ϕPA8. **Figure S6.** Growth phenotypes of MG1655 and the Stx2 lysogens in minimal medium supplemented with singe carbon sources. **Figure S7.** Bacterial size in growth experiments with minimal medium supplemented with single carbon sources. **Table S4.** Semi-quantitative determination of *stx2* copy number in MG1655::ϕO104. **Figure S8.** Kinetic measurements of the respiration potential of the strains using BIOLOG PM1 MicroPlate^TM^ Carbon Sources. **Table S5.** Statistical comparisons of the respiration potential of the strains using BIOLOG PM1 MicroPlate^TM^ Carbon Sources. **Table S6.** Normalized counts of sequencing reads mapped to A. ϕO104-encoded genes and B. ϕPA8-encoded genes. **Table S7.** Verifying the expression of *cI* and *cro* in MG1655 pWKS-*cI* and MG1655 pWKS-*cro*, respectively. **Figure S9.** Growth phenotypes of c*I* and *cro* expression in MG1655 in minimal medium supplemented with single carbon sources. **Figure S10.** Heatmap of the respiration potential of MG1655 pWKS30, MG1655 pWKS30-*cI* and MG1655 pWKS30-cro. **Table S8.** Primers used in this study. (DOCX 4959 kb)

## Data Availability

The sequencing data has been deposited in the NCBI’s Gene Expression Omnibus [77] and can be accessed through GEO Series accession number GSE126710. Additional information and data are available upon contacting the corresponding author.

## Abbreviations

*E. coli*: *Escherichia coli*
EAEC: Enteroaggregative *Escherichia coli*
EHEC: Enterohemorrhagic *Escherichia coli*
HC: Hemorrhagic colitis
HUS: Hemolytic uremic syndrome
LEE: Locus of enterocyte effacement
RNA-seq: RNA sequencing
RT-PCR: Reverse transcription polymerase chain reaction
Stx: Shiga toxin
