# Supplementary material for: Carriage of Shiga toxin phage profoundly affects Escherichia coli gene expression and carbon source utilization
Source: BMC Genomics. 2019 Jun 17;20:504. doi: 10.1186/s12864-019-5892-x (PMC6580645; doi:10.1186/s12864-019-5892-x)
Supplement: Supplementary file 3 — Table S1. Overview of sequenced and mapped reads. Figure S1. Principal component analysis (PCA). Table S2. Up- and downregulated genes in MG1655::ϕO104 in comparison to naive MG1655. Table S3. Up- and downregulated genes in MG1655::ϕPA8 in comparison to naive MG1655. A. Upregulated genes. B. Downregulated genes. Figure S2. Genome alignment of ϕO104 and ϕPA8. Figure S3. Interaction analysis of the genes found upregulated in the Stx2 lysogens. Figure S4. Stx2 phage carriage enhances FliC expression and motility. Figure S5. Interaction analysis of the genes found downregulated in MG1655::ϕPA8. Figure S6. Growth phenotypes of MG1655 and the Stx2 lysogens in minimal medium supplemented with singe carbon sources. Figure S7. Bacterial size in growth experiments with minimal medium supplemented with single carbon sources. Table S4. Semi-quantitative determination of stx2 copy number in MG1655::ϕO104. Figure S8. Kinetic measurements of the respiration potential of the strains using BIOLOG PM1 MicroPlateTM Carbon Sources. Table S5. Statistical comparisons of the respiration potential of the strains using BIOLOG PM1 MicroPlateTM Carbon Sources. Table S6. Normalized counts of sequencing reads mapped to A. ϕO104-encoded genes and B. ϕPA8-encoded genes. Table S7. Verifying the expression of cI and cro in MG1655 pWKS-cI and MG1655 pWKS-cro, respectively. Figure S9. Growth phenotypes of cI and cro expression in MG1655 in minimal medium supplemented with single carbon sources. Figure S10. Heatmap of the respiration potential of MG1655 pWKS30, MG1655 pWKS30-cI and MG1655 pWKS30-cro. Table S8. Primers used in this study. (DOCX 4959 kb) [file 12864_2019_5892_MOESM3_ESM.docx]

Carriage of Shiga toxin phage profoundly affects *E. coli* gene expression and carbon source utilization

Petya Berger, Ivan U. Kouzel, Michael Berger, Nadja Haarmann, Ulrich Dobrindt, Gerald B. Koudelka, Alexander Mellmann

Additional file 3

**Table S1. Overview of sequenced and mapped reads**

| **Libraries** | **MG1655** | | | **MG1655::φO104** | | | **MG1655::φPA8** | | |
| --- | --- | --- | --- | --- | --- | --- | --- | --- | --- |
|  | **1** | **2** | **3** | **1** | **2** | **3** | **1** | **2** | **3** |
| **No. of input reads used for alignment** | 14,368,118 | 11,989,302 | 10,555,764 | 19,218,706 | 16,443,487 | 19,271,772 | 13,868,451 | 10,121,932 | 10,779,077 |
| **No. of aligned reads** | 13,856,640 | 11,566,186 | 10,230,274 | 18,649,148 | 15,702,393 | 18,702,681 | 13,412,567 | 9,540,634 | 10,443,440 |
| **% of aligned reads** | 96.44 | 96.47 | 96.92 | 97.04 | 95.49 | 97.05 | 96.71 | 94.26 | 96.89 |
| **No. of aligned reads to MG1655 (U00096.3)** | 13,856,527 | 11,566,046 | 10,230,214 | 18,556,430 | 15,619,254 | 18,577,831 | 13,303,043 | 9,453,341 | 10,346,092 |
| **No. of aligned reads to φO104 (NC_018658.1*)** | 18 | 54 | 9 | 69,834 | 61,848 | 92,280 | 26,444 | 22,826 | 26,156 |
| **No. of aligned reads to φPA8 (KP682374.1)** | 95 | 86 | 51 | 22,884 | 21,292 | 32,570 | 83,080 | 64,466 | 71,192 |

*****Reads were mapped to the φO104 genome, which corresponds to the sequence from position 3,256,115 to 3,317,011 in NC_018658.1 (*E. coli* O104:H4 chromosome).


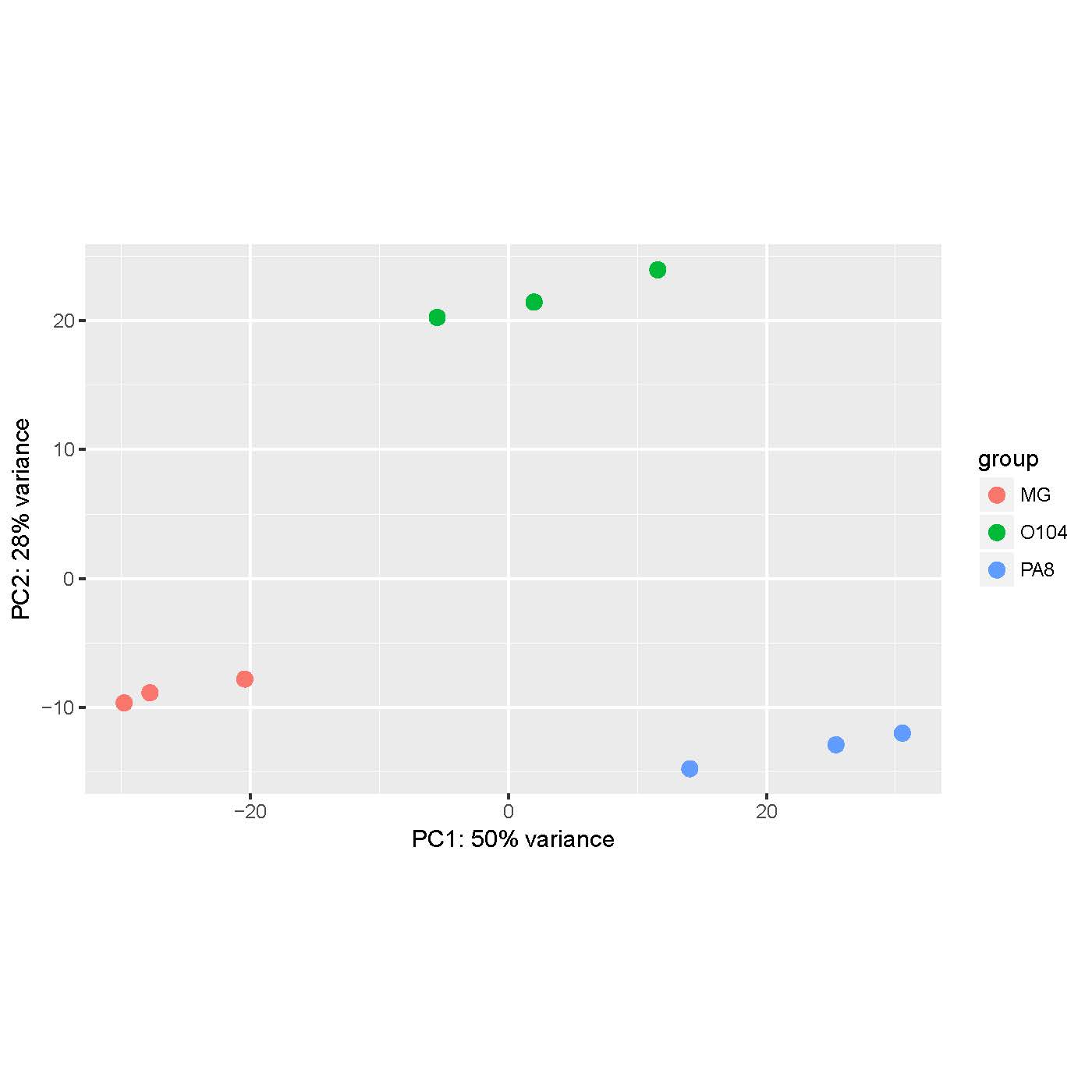


**Fig. S1. Principal component analysis (PCA).** The PCA is based on the gene expression data quantified with DESeq2. Each color represents a different strain (red=MG1655; green=MG1655::φO104; blue = MG1655::φPA8) and each dot represents a biological replicate.

**Table S2. Up- and downregulated genes in MG1655::φO104 in comparison to naive MG1655. A. Upregulated genes. B. Downregulated genes.** Genes with log2fold change > 0.58 (1.5 fold) and padj < 0.1 were considered. Genes are ranked based on their log2fold change in gene expression. The function of the genes was adapted from the EcoCyc *E. coli* Database. In the Overlap column genes are marked as follows: + genes found up- or downregulated in both MG1655::φO104 and MG1655::φPA8; +/- genes, which are found accordingly up- or downregulated in MG1655::φPA8 but below one of the thresholds applied in our analysis; - no overlap between MG1655::φO104 and MG1655::φPA8 datasets.

**A.**

| **Rank** | **Gene name** | **Gene function** | **log2Fold Change** | **padj** | **Overlap** |
| --- | --- | --- | --- | --- | --- |
| 1 | *yeeD* | putative sulfurtransferase YeeD | 3.76330596 | 0.0126302 | + |
| 2 | *yeeE* | inner membrane protein YeeE | 3.57820058 | 0.00613684 | + |
| 3 | *ydjN* | cystine/cysteine/sulfocysteine:cation symporter | 2.65292616 | 0.01944222 | + |
| 4 | *recN* | DNA repair protein RecN | 2.58029119 | 6.77E-33 | + |
| 5 | *umuD* | DNA polymerase V protein UmuD | 2.42802927 | 5.17E-16 | + |
| 6 | *sulA* | cell division inhibitor SulA | 2.38874775 | 1.01E-12 | + |
| 7 | *yebG* | DNA damage-inducible protein YebG | 2.21497236 | 4.89E-43 | + |
| 8 | *umuC* | DNA polymerase V catalytic protein | 2.21194078 | 3.52E-13 | + |
| 9 | *dinI* | DNA damage-inducible protein I | 2.12823072 | 7.63E-104 | + |
| 10 | *tisB* | membrane-depolarizing toxin TisB | 2.09720486 | 1.95E-34 | + |
| 11 | *recA* | DNA recombination/repair protein RecA | 1.90112186 | 4.26E-58 | + |
| 12 | *dinD* | DNA damage-inducible protein D | 1.81628599 | 0.00814718 | + |
| 13 | *ndh* | NADH:quinone oxidoreductase II | 1.77868158 | 0.00069611 | + |
| 14 | *yebF* | secreted protein YebF | 1.7566988 | 1.32E-59 | + |
| 15 | *recX* | RecA inhibitor RecX | 1.73321758 | 1.33E-05 | + |
| 16 | *ycfJ* | PF05433 family protein YcfJ | 1.61556132 | 6.28E-06 | + |
| 17 | *osmB* | osmotically-inducible lipoprotein OsmB | 1.58725337 | 3.41E-13 | + |
| 18 | *ydjM* | putative inner membrane protein regulated by LexA | 1.51770314 | 9.49E-14 | + |
| 19 | *dinB* | DNA polymerase IV | 1.51513625 | 6.28E-06 | + |
| 20 | *cho* | endonuclease of nucleotide excision repair | 1.41786942 | 4.93E-08 | + |
| 21 | *pdhR* | DNA-binding transcriptional dual regulator PdhR | 1.39353454 | 0.00557987 | + |
| 22 | *lexA* | DNA-binding transcriptional repressor LexA | 1.32811311 | 2.82E-06 | + |
| 23 | *xisE* | e14 prophage; putative excisionase | 1.322679 | 0.00014327 | + |
| 24 | *uvrA* | excision nuclease subunit A | 1.3056957 | 4.60E-16 | + |
| 25 | *symE* | toxic protein SymE | 1.24259592 | 0.00116445 | + |
| 26 | *croE* | e14 prophage; putative DNA-binding transcriptional regulator YmfT | 1.13956978 | 0.0671375 | + |
| 27 | *polB* | DNA polymerase II | 1.1224618 | 0.00238557 | + |
| 28 | *dinQ* | UV inducible membrane toxin DinQ | 1.09764609 | 4.81E-13 | + |
| 29 | *aceE* | pyruvate dehydrogenase E1 component | 1.05330673 | 0.0826497 | + |
| **Rank** | **Gene name** | **Gene function** | **log2Fold Change** | **padj** | **Overlap** |
| 30 | *ruvA* | Holliday junction branch migration complex subunit RuvA | 0.98779815 | 1.72E-12 | + |
| 31 | *ydeP* | putative oxidoreductase YdeP | 0.9829803 | 4.23E-06 | + |
| 32 | *ydcC* | conserved protein YdcC | 0.97167515 | 0.05269092 | +/- |
| 33 | *dinF* | DNA damage-inducible protein F | 0.95964165 | 6.45E-12 | + |
| 34 | *uvrB* | excision nuclease subunit B | 0.95686505 | 1.26E-09 | + |
| 35 | *flxA* | Qin prophage; protein FlxA | 0.95450423 | 2.37E-06 | + |
| 36 | *ypeC* | DUF2502 domain-containing protein YpeC | 0.94508337 | 7.96E-05 | + |
| 37 | *ymfL* | e14 prophage; uncharacterized protein YmfL | 0.93167623 | 0.00569702 | + |
| 38 | *yeaG* | protein kinase YeaG | 0.92543712 | 0.03416248 | +/- |
| 39 | *dinG* | ATP-dependent helicase | 0.86287215 | 0.00223219 | + |
| 40 | *ydeA* | L-arabinose exporter | 0.86051474 | 0.0808826 | - |
| 41 | *ruvB* | Holliday junction branch migration complex subunit RuvB | 0.84633492 | 2.33E-08 | + |
| 42 | *ackA* | acetate kinase | 0.81093517 | 0.05804685 | + |
| 43 | *pliG* | inhibitor of g-type lysozyme | 0.80325644 | 0.07019887 | +/- |
| 44 | *torS* | sensory histidine kinase TorS | 0.77040197 | 0.00069611 | - |
| 45 | *ymfJ* | e14 prophage; protein YmfJ | 0.76955124 | 0.02932043 | + |
| 46 | *cbpM* | chaperone modulator CbpM | 0.74574996 | 0.05952194 | - |
| 47 | *artI* | putative ABC transporter periplasmic binding protein ArtI | 0.74242746 | 2.49E-05 | + |
| 48 | *proP* | osmolyte:H+ symporter ProP | 0.74226028 | 0.00731359 | + |
| 49 | *asnA* | asparagine synthetase A | 0.74225269 | 0.03713956 | +/- |
| 50 | *ldhA* | D-lactate dehydrogenase | 0.74203552 | 0.06103743 | + |
| 51 | *ycjX* | DUF463 domain-containing protein YcjX | 0.71333275 | 0.00859314 | + |
| 52 | *cheW* | chemotaxis protein CheW | 0.6740986 | 4.93E-08 | + |
| 53 | *osmC* | osmotically inducible peroxiredoxin | 0.67159288 | 0.00354844 | + |
| 54 | *ybiB* | nonspecific DNA-binding protein YbiB | 0.66234949 | 0.05874308 | + |
| 55 | *ytfE* | iron-sulfur cluster repair protein YtfE | 0.65192196 | 0.02716376 | + |
| 56 | *yjbJ* | putative stress response protein | 0.64340775 | 0.02932043 | + |
| 57 | *ynjH* | DUF1496 domain-containing protein YnjH | 0.64270093 | 0.0058701 | + |
| 58 | *poxB* | pyruvate oxidase | 0.63859272 | 0.05071936 | - |
| 59 | *mscS* | small conductance mechanosensitive channel MscS | 0.63815138 | 0.01861063 | - |
| 60 | *uhpT* | hexose-6-phosphate:phosphate antiporter | 0.62027618 | 0.08886216 | + |
| 61 | *hdhA* | 7-a-hydroxysteroid dehydrogenase | 0.61183702 | 0.00212703 | - |
| 62 | *tar* | methyl-accepting chemotaxis protein Tar | 0.6007105 | 0.01050917 | + |
| 63 | *fliC* | flagellar filament structural protein | 0.58509861 | 0.00029318 | + |

**B.**

| **Rank** | **Gene name** | **Gene function** | **log2Fold Change** | **padj** | **Overlap** |
| --- | --- | --- | --- | --- | --- |
| 1 | *mglA* | D-galactose/methyl-galactoside ABC transporter ATP binding subunit | -4.00042408 | 1.19E-32 | + |
| 2 | *yjiY* | pyruvate:H+ symporter | -3.9124322 | 5.32E-18 | + |
| 3 | *galP* | galactose:H+ symporter | -3.67995697 | 1.22E-24 | + |
| 4 | *mglC* | D-galactose/methyl-galactoside ABC transporter membrane subunit | -3.50336808 | 2.65E-16 | + |
| 5 | *dsdX* | D-serine transporter | -3.49104282 | 8.90E-39 | + |
| 6 | *mglB* | D-galactose/methyl-galactoside ABC transporter periplasmic binding protein | -3.40531482 | 4.61E-12 | + |
| 7 | *nanC* | *N*-acetylneuraminic acid outer membrane channel | -3.10341134 | 0.01718801 | +/- |
| 8 | *nanS* | putative 9-*O*-acetyl-*N*-acetylneuraminate esterase | -3.09304668 | 0.00092708 | + |
| 9 | *lldP* | (R)-lactate/(S)-lactate/glycolate:H+ symporter LldP | -3.0869776 | 3.21E-05 | + |
| 10 | *galS* | DNA-binding transcriptional dual regulator GalS | -2.98229046 | 5.30E-09 | + |
| 11 | *galT* | galactose-1-phosphate uridylyltransferase | -2.71687147 | 3.30E-09 | + |
| 12 | *nanM* | *N*-acetylneuraminate mutarotase | -2.62972546 | 0.0080356 | +/- |
| 13 | *lldR* | DNA-binding transcriptional dual regulator LldR ('lactate regulator') | -2.60955994 | 1.06E-09 | + |
| 14 | *nanT* | N-acetylneuraminate:H^+^ symporter | -2.36663665 | 2.38E-05 | + |
| 15 | *galE* | UDP-glucose 4-epimerase | -2.22914713 | 3.43E-06 | + |
| 16 | *yjhB* | putative sialic acid transporter | -2.09878657 | 0.00182956 | + |
| 17 | *dsdA* | D-serine ammonia-lyase | -2.08450889 | 0.00169815 | + |
| 18 | *nanE* | putative *N*-acetylmannosamine-6-phosphate epimerase | -1.91538309 | 7.49E-06 | + |
| 19 | *nanK* | *N*-acetylmannosamine kinase | -1.84764066 | 0.00010357 | + |
| 20 | *lldD* | L-lactate dehydrogenase | -1.74096685 | 5.58E-05 | + |
| 21 | *yhcH* | DUF386 domain-containing protein YhcH | -1.70918785 | 0.00444985 | + |
| 22 | *dctA* | C4 dicarboxylate/orotate:H^+^ symporter | -1.64187292 | 0.01783913 | + |
| 23 | *lamB* | maltose outer membrane porin / phage lambda receptor protein | -1.51675575 | 0.00029099 | + |
| 24 | *glpQ* | glycerophosphoryl diester phosphodiesterase, periplasmic | -1.45972393 | 0.00562545 | + |
| 25 | *nanA* | *N*-acetyl-β-neuraminate lyase / *N*-acetylneuraminate lyase | -1.4496376 | 0.00019083 | + |
| 26 | *yqeF* | putative acyltransferase | -1.40526565 | 0.07976585 | +/- |
| 27 | *malK* | maltose ABC transporter ATP binding subunit | -1.38910011 | 0.00613684 | + |
| 28 | *malF* | maltose ABC transporter membrane subunit MalF | -1.38146086 | 5.27E-05 | + |
| **Rank** | **Gene name** | **Gene function** | **log2Fold Change** | **padj** | **Overlap** |
| 29 | *galK* | Galactokinase | -1.36120327 | 4.45E-20 | + |
| 30 | *putA* | fused DNA-binding transcriptional repressor / proline dehydrogenase / 1-pyrroline-5-carboxylate dehydrogenase PutA | -1.32298789 | 0.01869117 | + |
| 31 | *entC* | isochorismate synthase EntC | -1.30977487 | 0.07406907 | + |
| 32 | *fiu* | putative iron siderophore outer membrane transporter | -1.29968638 | 0.05874308 | +/- |
| 33 | *malE* | maltose ABC transporter periplasmic binding protein | -1.25209294 | 0.0058701 | + |
| 34 | *yjhC* | KpLE2 phage-like element; putative oxidoreductase YjhC | -1.16580026 | 0.01370284 | - |
|  |  |  |  |  |  |
| 36 | *malG* | maltose ABC transporter membrane subunit MalG | -1.13124199 | 1.06E-05 | + |
| 37 | *srlD* | sorbitol-6-phosphate 2-dehydrogenase | -1.09588684 | 5.38E-06 | + |
| 38 | *yihN* | putative transporter YihN | -1.07606466 | 0.01042232 | + |
| 39 | *malM* | maltose regulon periplasmic protein | -1.06102873 | 0.00616011 | + |
| 40 | *cytR* | DNA-binding transcriptional repressor CytR | -0.99498145 | 0.00156997 | + |
| 41 | *srlB* | sorbitol-specific PTS enzyme IIA component | -0.97802775 | 5.13E-06 | + |
| 42 | *acs* | acetyl-CoA synthetase (AMP-forming) | -0.94324643 | 0.06765649 | + |
| 43 | *nrdF* | ribonucleoside-diphosphate reductase 2, β subunit dimer | -0.93567054 | 1.09E-06 | + |
| 44 | *galM* | galactose-1-epimerase | -0.86831804 | 2.73E-09 | + |
| 45 | *nrdE* | ribonucleoside-diphosphate reductase 2, α subunit dimer | -0.85113437 | 0.00841331 | + |
| 46 | *ebgA* | evolved β-D-galactosidase subunit α | -0.81566123 | 0.00680891 | + |
| 47 | *dsdC* | DNA-binding transcriptional dual regulator DsdC | -0.79911649 | 1.79E-06 | + |
| 48 | *glpK* | glycerol kinase | -0.78084854 | 0.09924998 | +/- |
| 49 | *nrdI* | dimanganese-tyrosyl radical cofactor maintenance flavodoxin NrdI | -0.7704128 | 0.05319929 | + |
| 50 | *frwC* | putative PTS enzyme IIC component FrwC | -0.75852972 | 0.04829216 | + |
| 51 | *gatA* | galactitol-specific PTS enzyme IIA component | -0.74159801 | 0.03380873 | - |
| 52 | *gatB* | galactitol-specific PTS enzyme IIB component | -0.73169141 | 7.40E-12 | +/- |
| 53 | *fumA* | fumarase A | -0.72799226 | 0.04829216 | + |
| 54 | *glpD* | aerobic glycerol 3-phosphate dehydrogenase | -0.72744315 | 0.00315346 | +/- |
| 55 | *ytfF* | inner membrane protein YtfF | -0.71550054 | 0.05490967 | + |
| 56 | *mhpR* | DNA-binding transcriptional activator MhpR | -0.6927238 | 0.09924998 | + |
| 57 | *fecE* | ferric citrate ABC transporter ATP binding subunit | -0.68664554 | 0.00058924 | +/- |
| 58 | *fecC* | ferric citrate ABC transporter membrane subunit FecC | -0.68268404 | 0.00557987 | +/- |
| 59 | *ytfQ* | galactofuranose ABC transporter periplasmic binding protein | -0.6649765 | 0.09667764 | - |
| 60 | *maeB* | malate dehydrogenase | -0.64743265 | 0.04829216 | + |
| 61 | *fucI* | L-fucose isomerase | -0.64583008 | 0.00349311 | + |
| 62 | *ompF* | outer membrane porin F | -0.63380216 | 0.00029318 | - |
| 63 | *nuoB* | NADH:quinone oxidoreductase subunit B | -0.62597099 | 0.00047782 | +/- |
| 64 | *treA* | periplasmic trehalase | -0.61860298 | 0.0820266 | + |
| **Rank** | **Gene name** | **Gene function** | **log2Fold Change** | **padj** | **Overlap** |
| 65 | *fecD* | ferric citrate ABC transporter membrane subunit FecD | -0.61192499 | 0.03438198 | - |
| 66 | *nuoA* | NADH:quinone oxidoreductase subunit A | -0.60713223 | 0.0058701 | +/- |
| 67 | *insL* | IS186/IS421 transposase (AAC73683.1) | -0.59665442 | 0.02932043 | + |
| 68 | *insL* | IS186/IS421 transposase (AAC75453.2) | -0.58864462 | 0.0826497 | + |
| 69 | *nuoC* | NADH:quinone oxidoreductase subunit CD | -0.58562076 | 0.00276437 | + |

**Table S3. Up- and downregulated genes in MG1655::φPA8 in comparison to naive MG1655. A. Upregulated genes. B. Downregulated genes.** Genes with log2fold change > 0.58 (1.5 fold) and padj < 0.1 were considered. Genes are ranked based on their log2fold change in gene expression. The gene function of the genes was adapted from the EcoCyc *E. coli* Database. In the Overlap column genes are marked as follows: + genes found up- or downregulated in both MG1655::φO104 and MG1655::φPA8; +/- genes, which are found accordingly up- or downregulated in MG1655::φO104 but below one of the thresholds applied in our analysis; - no overlap between MG1655::φO104 and MG1655::φPA8 datasets.

**A.**

| **Rank** | **Gene name** | **Gene function** | **log2Fold**  **Change** | **padj** | **Overlap** |
| --- | --- | --- | --- | --- | --- |
| 1 | *yfdR* | CPS-53 (KpLE1) prophage; 5'-deoxynucleotidase | 5.674478 | 1.29E-05 | +/- |
| 2 | *yeeE* | inner membrane protein YeeE | 4.351972 | 0.00025084 | + |
| 3 | *cysP* | thiosulfate/sulfate ABC transporter periplasmic binding protein CysP | 4.018729 | 0.00222638 | +/- |
| 4 | *yeeD* | putative sulfurtransferase YeeD | 3.886153 | 0.00858984 | + |
| 5 | *ydjN* | cystine/cysteine/sulfocysteine:cation symporter | 3.38041 | 0.00065267 | + |
| 6 | *cysW* | sulfate/thiosulfate ABC transporter inner membrane subunit CysW | 3.234286 | 0.06127675 | +/- |
| 7 | *adiY* | DNA-binding transcriptional activator AdiY | 3.109544 | 0.09257359 | +/- |
| 8 | *yfdS* | CPS-53 (KpLE1) prophage; protein YfdS | 3.00554 | 6.41E-07 | - |
| 9 | *recN* | DNA repair protein RecN | 2.798338 | 1.04E-38 | + |
| 10 | *cysD* | sulfate adenylyltransferase subunit 2 | 2.756796 | 0.04653933 | +/- |
| 11 | *cysU* | sulfate/thiosulfate ABC transporter inner membrane subunit CysU | 2.738826 | 0.05086605 | +/- |
| 12 | *cysJ* | sulfite reductase, flavoprotein subunit | 2.726116 | 0.01050624 | +/- |
| 13 | *umuD* | DNA polymerase V protein UmuD | 2.670616 | 2.90E-19 | + |
| 14 | *sulA* | cell division inhibitor SulA | 2.63484 | 1.83E-15 | + |
| 15 | *dinI* | DNA damage-inducible protein I | 2.428737 | 4.91E-135 | + |
| 16 | *tisB* | membrane-depolarizing toxin TisB | 2.422759 | 4.56E-46 | + |
| 17 | *yebG* | DNA damage-inducible protein YebG | 2.370436 | 2.59E-49 | + |
| 18 | *umuC* | DNA polymerase V catalytic protein | 2.298638 | 3.79E-14 | + |
| 19 | *dinD* | DNA damage-inducible protein D | 2.220269 | 0.00036114 | + |
| 20 | *cysK* | cysteine synthase A | 2.186646 | 0.04249849 | +/- |
| 21 | *sbmC* | DNA gyrase inhibitor | 2.150216 | 0.03303208 | +/- |
| 22 | *recA* | DNA recombination/repair protein RecA | 1.964711 | 5.13E-62 | + |
| 23 | *yebF* | secreted protein YebF | 1.869837 | 4.62E-67 | + |
| 24 | *recX* | RecA inhibitor RecX | 1.764511 | 9.79E-06 | + |
| 25 | *crfC* | clamp-binding sister replication fork colocalization protein | 1.735145 | 0.08114651 | +/- |
| 26 | *ndh* | NADH:quinone oxidoreductase II | 1.718602 | 0.00111254 | + |
| 27 | *xisE* | e14 prophage; putative excisionase | 1.700378 | 1.78E-07 | + |
| 28 | *dinB* | DNA polymerase IV | 1.648026 | 5.46E-07 | + |
| 29 | *yfdT* | CPS-53 (KpLE1) prophage; protein YfdT | 1.606563 | 0.01309803 | - |
| 30 | *ydjM* | putative inner membrane protein regulated by LexA | 1.60498 | 3.63E-15 | + |
| **Rank** | **Gene name** | **Gene function** | **log2Fold**  **Change** | **padj** | **Overlap** |
| 31 | *croE* | e14 prophage; putative DNA-binding transcriptional regulator YmfT | 1.543305 | 0.00241206 | + |
| 32 | *osmB* | osmotically-inducible lipoprotein OsmB | 1.539847 | 4.26E-12 | + |
| 33 | *cho* | endonuclease of nucleotide excision repair | 1.512803 | 4.90E-09 | + |
| 34 | *symE* | toxic protein SymE | 1.511843 | 3.31E-05 | + |
| 35 | *yncG* | putative glutathione *S*-transferase YncG | 1.505436 | 0.04351493 | +/- |
| 36 | *lexA* | DNA-binding transcriptional repressor LexA | 1.485726 | 7.00E-08 | + |
| 37 | *ycfJ* | PF05433 family protein YcfJ | 1.484325 | 8.59E-05 | + |
| 38 | *yfdQ* | CPS-53 (KpLE1) prophage; protein YfdQ | 1.378692 | 1.49E-05 | - |
| 39 | *ymfL* | e14 prophage; uncharacterized protein YmfL | 1.312474 | 8.99E-06 | + |
| 40 | *ydiH* | protein YdiH | 1.304302 | 0.01590813 | +/- |
| 41 | *dinQ* | UV inducible membrane toxin DinQ | 1.300174 | 2.30E-18 | + |
| 42 | *uvrA* | excision nuclease subunit A | 1.277739 | 2.67E-15 | + |
| 43 | *pdhR* | DNA-binding transcriptional dual regulator PdhR | 1.236083 | 0.02147231 | + |
| 44 | *polB* | DNA polymerase II | 1.233228 | 0.00046886 | + |
| 45 | *flxA* | Qin prophage; protein FlxA | 1.208421 | 2.61E-10 | + |
| 46 | *aceE* | pyruvate dehydrogenase E1 component | 1.195172 | 0.02907569 | + |
| 47 | *yqiJ* | putative inner membrane protein | 1.12486 | 0.07759688 | +/- |
| 48 | *ydfO* | Qin prophage; protein YdfO | 1.121727 | 0.01324842 | +/- |
| 49 | *ypeC* | DUF2502 domain-containing protein YpeC | 1.116082 | 1.82E-06 | + |
| 50 | *sra* | 30S ribosomal subunit protein S22 | 1.061321 | 0.08137272 | +/- |
| 51 | *dinF* | DNA damage-inducible protein F | 1.058956 | 2.96E-14 | + |
| 52 | *yfdP* | CPS-53 (KpLE1) prophage; protein YfdP | 1.047853 | 0.06897058 | - |
| 53 | *dinG* | ATP-dependent helicase | 1.024554 | 9.28E-05 | + |
| 54 | *uvrB* | excision nuclease subunit B | 1.018119 | 7.92E-11 | + |
| 55 | *uhpT* | hexose-6-phosphate:phosphate antiporter | 0.996739 | 0.00022743 | + |
| 56 | *iraP* | anti-adaptor protein for σ^S^ stabilization | 0.993961 | 0.00082887 | - |
| 57 | *ymfJ* | e14 prophage; protein YmfJ | 0.985332 | 0.00134598 | + |
| 58 | *ynjH* | DUF1496 domain-containing protein YnjH | 0.975175 | 8.42E-07 | + |
| 59 | *ruvA* | Holliday junction branch migration complex subunit RuvA | 0.942025 | 4.75E-11 | + |
| 60 | *ackA* | acetate kinase | 0.926324 | 0.01559613 | + |
| 61 | *ydeP* | putative oxidoreductase YdeP | 0.902281 | 9.08E-05 | + |
| 62 | *ruvB* | Holliday junction branch migration complex subunit RuvB | 0.872362 | 1.03E-08 | + |
| 63 | *proP* | osmolyte:H^+^ symporter ProP | 0.825571 | 0.00163838 | + |
| 64 | *tar* | methyl-accepting chemotaxis protein Tar | 0.815216 | 7.14E-05 | + |
| 65 | *pmrR* | putative bitopic inner membrane protein | 0.801144 | 0.04351493 | - |
| 66 | *fliC* | flagellar filament structural protein | 0.784288 | 9.71E-08 | + |
| 67 | *pptA* | putative 4-oxalocrotonate tautomerase (4-OT) | 0.782597 | 0.00115892 | - |
| 68 | *ybiB* | nonspecific DNA-binding protein YbiB | 0.762988 | 0.01524864 | + |
| 69 | *ybgS* | conserved protein YbgS | 0.742728 | 0.03140815 | +/- |
| 70 | *ogrK* | prophage P2 late control protein OgrK | 0.732808 | 0.00081321 | - |
| 71 | *yhjH* | c-di-GMP phosphodiesterase PdeH | 0.703083 | 0.05104364 | - |
| 72 | *ldhA* | D-lactate dehydrogenase | 0.702003 | 0.09107364 | + |
| 73 | *yqaE* | Pmp3 family protein | 0.701792 | 0.07888736 | +/- |
| 74 | *ydcY* | DUF2526 domain-containing protein YdcY | 0.698391 | 0.04673554 | - |
| 75 | *artI* | putative ABC transporter periplasmic binding protein ArtI | 0.69308 | 0.00014729 | + |
| 76 | *osmC* | osmotically inducible peroxiredoxin | 0.692161 | 0.00246007 | + |
| 77 | *yjbJ* | putative stress response protein | 0.675743 | 0.01992373 | + |
| 78 | *cheW* | chemotaxis protein CheW | 0.674919 | 6.22E-08 | + |
| **Rank** | **Gene name** | **Gene function** | **log2Fold**  **Change** | **padj** | **Overlap** |
| 79 | *mtr* | tryptohan:H^+^ symporter Mtr | 0.673818 | 0.02062005 | - |
| 80 | *motA* | motility protein A | 0.667784 | 0.04545493 | - |
| 81 | *ycjX* | DUF463 domain-containing protein YcjX | 0.665825 | 0.02266991 | + |
| 82 | *ydhR* | putative monooxygenase YdhR | 0.664152 | 0.04673554 | +/- |
| 83 | *tap* | methyl-accepting chemotaxis protein Tap | 0.644294 | 0.00036114 | +/- |
| 84 | *ygaU* | K^+^ binding protein | 0.643409 | 0.09107364 | +/- |
| 85 | *motB* | motility protein B | 0.642795 | 0.07296096 | - |
| 86 | *ytfE* | iron-sulfur cluster repair protein YtfE | 0.642056 | 0.04011956 | + |
| 87 | *modA* | molybdate ABC transporter periplasmic binding protein | 0.627624 | 0.07704802 | - |
| 88 | *ygdI* | DUF903 domain-containing lipoprotein YgdI | 0.623121 | 0.05301173 | - |
| 89 | *yfcI* | recombination-promoting nuclease RpnB | 0.602406 | 0.02062005 | - |
| 90 | *dosC* | diguanylate cyclase DosC | 0.592118 | 0.01257265 | - |
| 91 | *yihD* | conserved protein YihD | 0.585231 | 0.02187325 | - |
| 92 | *cheZ* | chemotaxis protein CheZ | 0.584823 | 8.05E-05 | +/- |
| 93 | *cheR* | chemotaxis protein methyltransferase | 0.582671 | 0.004986 | - |
| 94 | *argG* | argininosuccinate synthetase | 0.582317 | 0.00049302 | +/- |

**B.**

| **Rank** | **Gene name** | **Gene function** | **log2Fold**  **Change** | **padj** | **Overlap** |
| --- | --- | --- | --- | --- | --- |
| 1 | *yjiY* | pyruvate:H^+^ symporter | -4.39901 | 8.72E-23 | + |
| 2 | *mglA* | D-galactose/methyl-galactoside ABC transporter ATP binding subunit | -4.18225 | 7.00E-35 | + |
| 3 | *mglC* | D-galactose/methyl-galactoside ABC transporter membrane subunit | -3.72605 | 2.83E-18 | + |
| 4 | *galP* | galactose:H^+^ symporter | -3.66514 | 3.79E-24 | + |
| 5 | *mglB* | D-galactose/methyl-galactoside ABC transporter periplasmic binding protein | -3.59797 | 1.96E-13 | + |
| 6 | *lldP* | (R)-lactate/(S)-lactate/glycolate:H^+^ symporter LldP | -3.30032 | 6.11E-06 | + |
| 7 | *dsdX* | D-serine transporter | -3.28007 | 3.50E-33 | + |
| 8 | *galS* | DNA-binding transcriptional dual regulator GalS | -2.88893 | 3.27E-08 | + |
| 9 | *lldR* | DNA-binding transcriptional dual regulator LldR | -2.63123 | 9.60E-10 | + |
| 10 | *galT* | galactose-1-phosphate uridylyltransferase | -2.61902 | 1.93E-08 | + |
| 11 | *dsdA* | D-serine ammonia-lyase | -2.25943 | 0.0003839 | + |
| 12 | *yhdV* | lipoprotein YhdV | -2.19133 | 8.89E-05 | +/- |
| 13 | *galE* | UDP-glucose 4-epimerase | -2.15439 | 8.99E-06 | + |
| 14 | *nanS* | putative 9-*O*-acetyl-*N*-acetylneuraminate esterase | -2.13575 | 0.06823597 | + |
| 15 | *nanT* | *N*-acetylneuraminate:H^+^ symporter | -2.03855 | 0.00053557 | + |
| 16 | *lldD* | L-lactate dehydrogenase | -1.93559 | 4.08E-06 | + |
| 17 | *nanK* | *N*-acetylmannosamine kinase | -1.88869 | 6.68E-05 | + |
| 18 | *nanE* | putative *N*-acetylmannosamine-6-phosphate epimerase | -1.86612 | 1.53E-05 | + |
| 19 | *dhaL* | dihydroxyacetone kinase subunit L | -1.80708 | 0.0077899 | +/- |
| 20 | *yhcH* | DUF386 domain-containing protein YhcH | -1.7408 | 0.00304338 | + |
| 21 | *dhaM* | dihydroxyacetone kinase subunit M/protein-lysine deacetylase | -1.71474 | 0.00858179 | +/- |
| 22 | *dhaK* | dihydroxyacetone kinase subunit K | -1.69934 | 0.00700087 | +/- |
| 23 | *lamB* | maltose outer membrane porin / phage lambda receptor protein | -1.51097 | 0.00030061 | + |
| 24 | *dctA* | C4 dicarboxylate/orotate:H^+^ symporter | -1.46308 | 0.05354566 | + |
| 25 | *yjhB* | putative sialic acid transporter | -1.43609 | 0.09841804 | + |
| 26 | *fes* | enterochelin esterase | -1.41125 | 0.09383458 | +/- |
| 27 | *nrdE* | ribonucleoside-diphosphate reductase 2 subunit &alpha; | -1.35564 | 6.79E-07 | + |
| 28 | *malK* | maltose ABC transporter ATP binding subunit | -1.35393 | 0.00838106 | + |
| 29 | *nanA* | *N*-acetylneuraminate lyase | -1.3495 | 0.00066773 | + |
| 30 | *galK* | galactokinase | -1.33307 | 4.97E-19 | + |
| 31 | *entC* | isochorismate synthase EntC | -1.32572 | 0.06992568 | + |
| 32 | *putA* | fused DNA-binding transcriptional repressor / proline dehydrogenase / 1-pyrroline-5-carboxylate dehydrogenase PutA | -1.30845 | 0.02134318 | + |
| 33 | *malF* | maltose ABC transporter membrane subunit MalF | -1.27308 | 0.00031506 | + |
| 34 | *nrdH* | glutaredoxin-like protein | -1.20909 | 0.00031506 | +/- |
| 35 | *malE* | maltose ABC transporter periplasmic binding protein | -1.20628 | 0.00890309 | + |
| **Rank** | ***Gene name*** | **Gene function** | **log2Fold**  **Change** | **padj** | **Overlap** |
| 36 | *fadB* | dodecenoyl-CoA &delta;-isomerase, enoyl-CoA hydratase, 3-hydroxybutyryl-CoA epimerase, 3-hydroxyacyl-CoA dehydrogenase | -1.20088 | 0.06016182 | +/- |
| 37 | *cirA* | ferric dihyroxybenzoylserine outer membrane transporter | -1.19411 | 0.0492042 | + |
| 38 | *srlD* | sorbitol-6-phosphate 2-dehydrogenase | -1.18777 | 5.13E-07 | + |
| 39 | *nrdI* | dimanganese-tyrosyl radical cofactor maintenance flavodoxin NrdI | -1.15185 | 0.00031319 | + |
| 40 | *malG* | maltose ABC transporter membrane subunit MalG | -1.15166 | 1.31E-05 | + |
| 41 | *yihN* | putative transporter YihN | -1.1285 | 0.00793923 | + |
| 42 | *srlB* | sorbitol-specific PTS enzyme IIA component | -1.12332 | 9.44E-08 | + |
| 43 | *acs* | acetyl-CoA synthetase (AMP-forming) | -1.12074 | 0.01668362 | + |
| 44 | *rbsK* | Ribokinase | -1.11308 | 0.00274953 | +/- |
| 45 | *glpQ* | glycerophosphoryl diester phosphodiesterase | -1.11065 | 0.08073495 | + |
| 46 | *malM* | maltose regulon periplasmic protein | -1.07157 | 0.00565932 | + |
| 47 | *nrdF* | ribonucleoside-diphosphate reductase 2 subunit &beta; | -1.03434 | 8.34E-08 | + |
| 48 | *frwC* | putative PTS enzyme IIC component FrwC | -0.98757 | 0.0070234 | + |
| 49 | *fumA* | fumarase A | -0.9514 | 0.00198726 | + |
| 50 | *cytR* | DNA-binding transcriptional repressor CytR | -0.93472 | 0.00372241 | + |
| 51 | *galM* | galactose-1-epimerase | -0.93281 | 1.19E-10 | + |
| 52 | *ugpB* | *sn*-glycerol 3-phosphate ABC transporter periplasmic binding protein | -0.90831 | 0.08279671 | +/- |
| 53 | *ebgA* | evolved b-D-galactosidase subunit a; | -0.90237 | 0.00261486 | + |
| 54 | *rbsB* | ribose ABC transporter periplasmic binding protein | -0.88915 | 0.00612389 | +/- |
| 55 | *fucI* | L-fucose isomerase | -0.86528 | 3.44E-05 | + |
| 56 | *frwB* | putative PTS enzyme IIB component FrwB | -0.86482 | 0.04916737 | - |
| 57 | *dsdC* | DNA-binding transcriptional dual regulator DsdC | -0.84904 | 8.29E-07 | + |
| 58 | *maeB* | malate dehydrogenase | -0.77398 | 0.00708987 | + |
| 59 | *rbsD* | D-ribose pyranase | -0.77394 | 0.06313752 | - |
| 60 | *uxuB* | D-mannonate oxidoreductase | -0.77158 | 0.00185398 | +/- |
| 61 | *gntP* | fructuronate transporter | -0.76239 | 0.05375381 | +/- |
| 62 | *sufA* | iron-sulfur cluster insertion protein SufA | -0.75174 | 0.08640643 | + |
| 63 | *fecR* | regulator for fec operon, periplasmic | -0.75115 | 0.09825973 | +/- |
| 64 | *sufC* | Fe-S cluster scaffold complex subunit SufC | -0.73443 | 0.03430641 | - |
| 65 | *mhpR* | DNA-binding transcriptional activator MhpR | -0.72478 | 0.08884614 | + |
| 66 | *ytfF* | inner membrane protein YtfF | -0.69423 | 0.07889867 | + |
| 67 | *treA* | periplasmic trehalase | -0.68496 | 0.05375381 | + |
| 68 | *uxuA* | D-mannonate dehydratase | -0.6491 | 0.07888736 | +/- |
| 69 | *grpE* | nucleotide exchange factor GrpE | -0.64626 | 0.05947729 | +/- |
| 70 | *nuoC* | NADH:quinone oxidoreductase subunit CD | -0.64549 | 0.00050581 | + |
| 71 | *insL* | IS186/IS421 transposase (AAC75453.2) | -0.59543 | 0.07842895 | + |
| 72 | *insL* | IS186/IS421 transposase (AAC73683.1) | -0.5903 | 0.03525187 | + |

**Fig. S2. Genome alignment of φO104 and φPA8.** The genomes of φO104 and φPA8 were aligned using Mauve and progressive genome alignment. The four color blocks (red, yellow, green, blue) indicate the collinear and homologues regions among the genomes, with the boundaries being the breakpoints of genomic rearrangement.

**φO104**

**φPA8**

**Fig. S3. Interaction analysis of the genes found upregulated in the Stx2 lysogens.** The network summarizes the predicted associations for the proteins encoded by the upregulates genes detected in our analysis. The network nodes are proteins and the edges represent the predicted functional associations. The thickness of the line indicates the degree of confidence prediction of the interaction. The network was created using STRING 11.0.

**A. Upregulated genes in MG1655::φO104.**

**
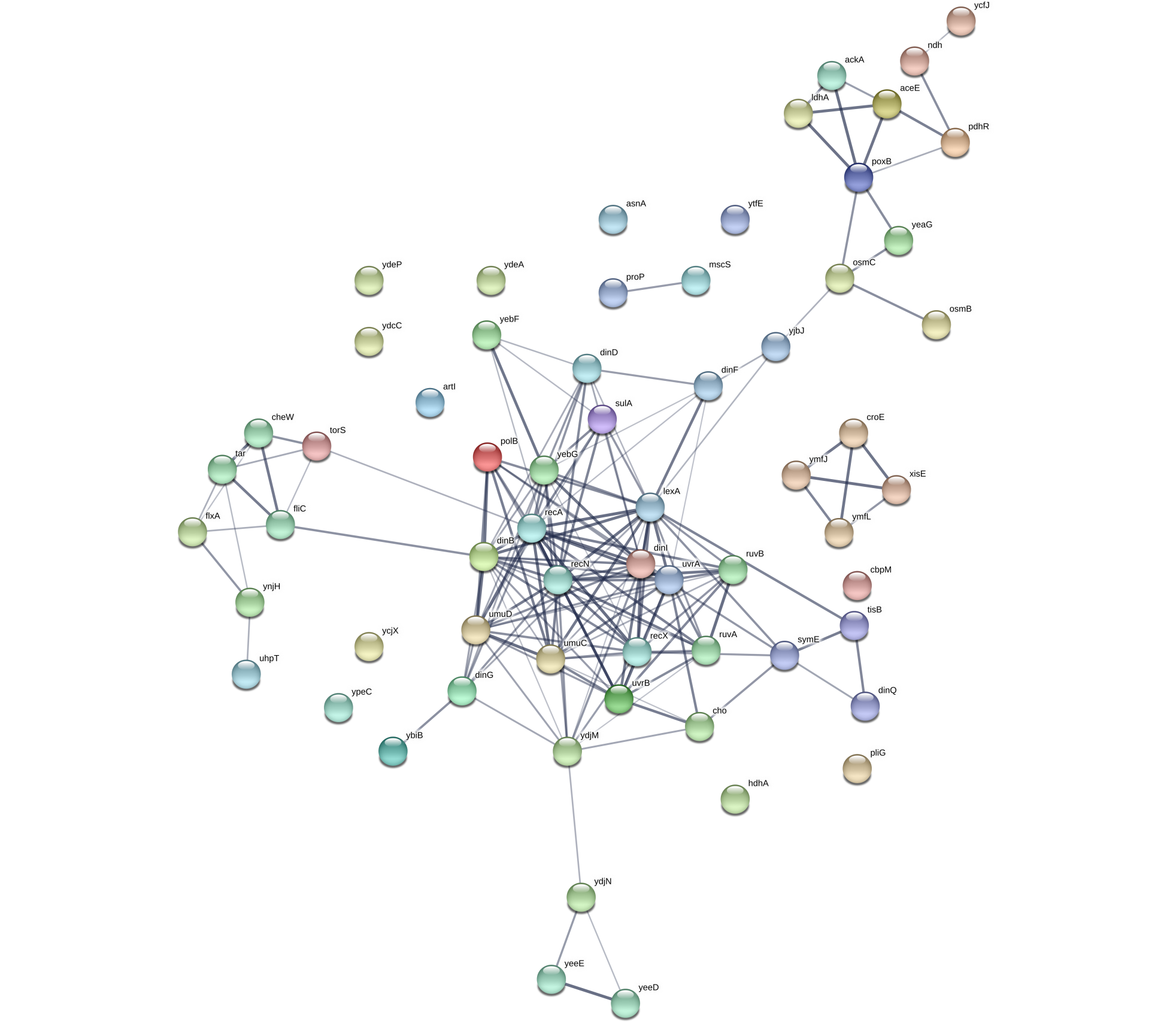
**

**B. Upregulated genes in MG1655::φPA8**

**
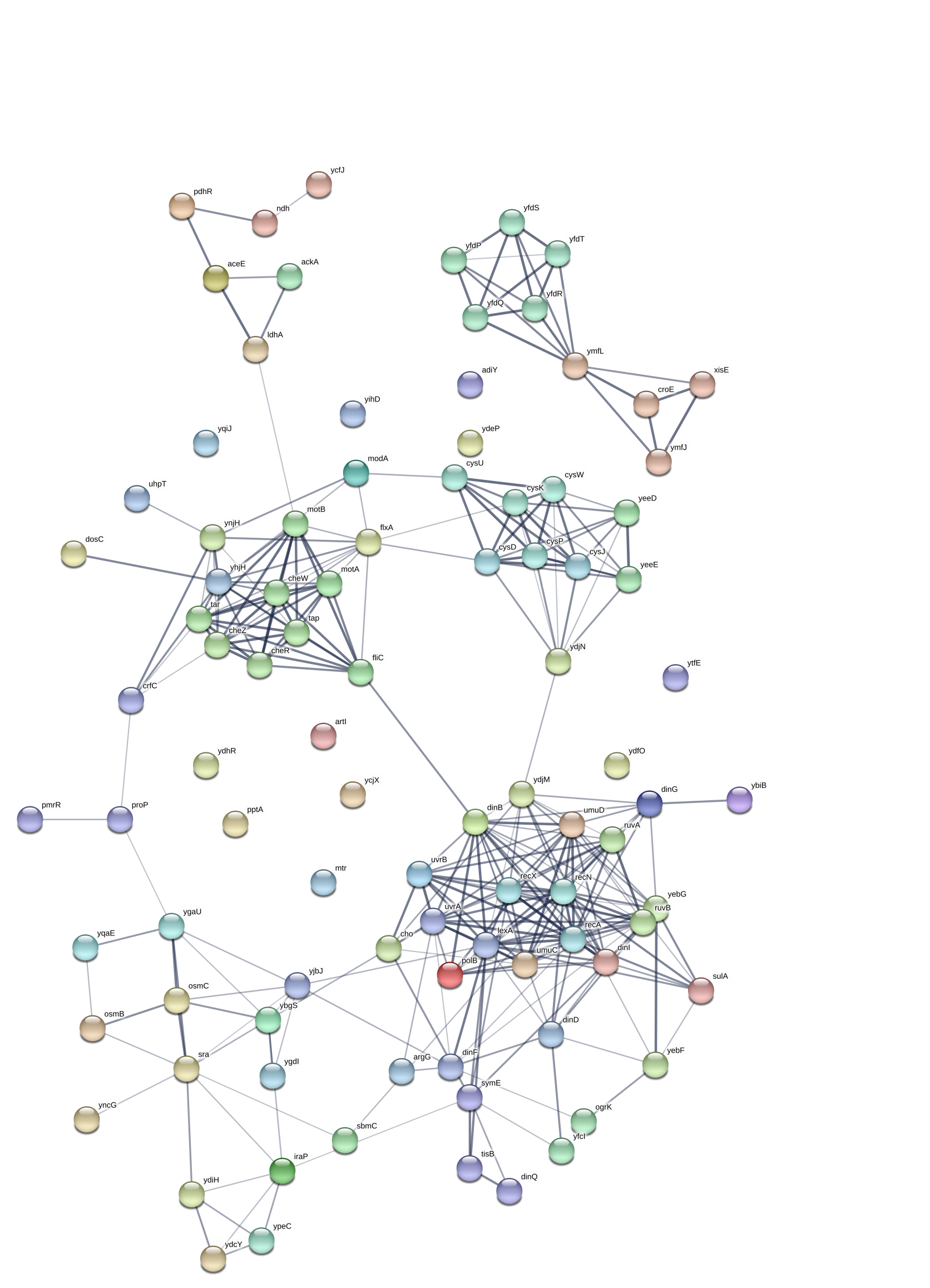
**

**Fig. S4. Stx2 phage carriage enhances FliC expression and motility. A. FliC detection in total protein preparations with semi-quantitative Western blot.** The expected FliC band is marked by arrows. Marker (M) = Protein Marker IV (Peqlab). MG1655 Δ*fliC* was used here as a negative control. MG1655::φO104 expresses 1.8 fold higher levels FliC protein (p = 0.01919). **B. Swimming motility.** The swimming phenotype of the strains was evaluated on motility 0.3% agar plates. Below the photograph the diameter (d = mean and standard deviation of three biological replicates) of the swimming zones is given. MG1655::φO104 displays significantly increased swimming motility (p = 0.021)

**
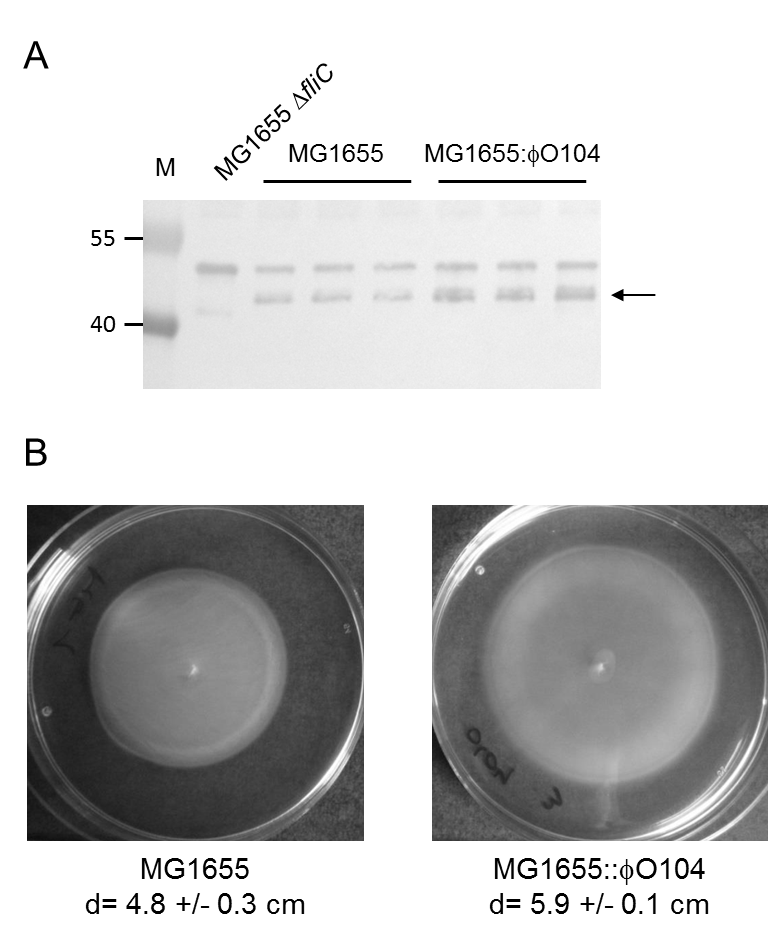
**

**Fig. S5. Interaction analysis of the genes found downregulated in MG1655::φPA8.** The network summarizes the predicted associations for the proteins encoded by the downregulated genes detected in our analysis. The network nodes are proteins and the edges represent the predicted functional associations. The thickness of the line indicates the degree of confidence prediction of the interaction. The network was created using STRING 11.0.

**
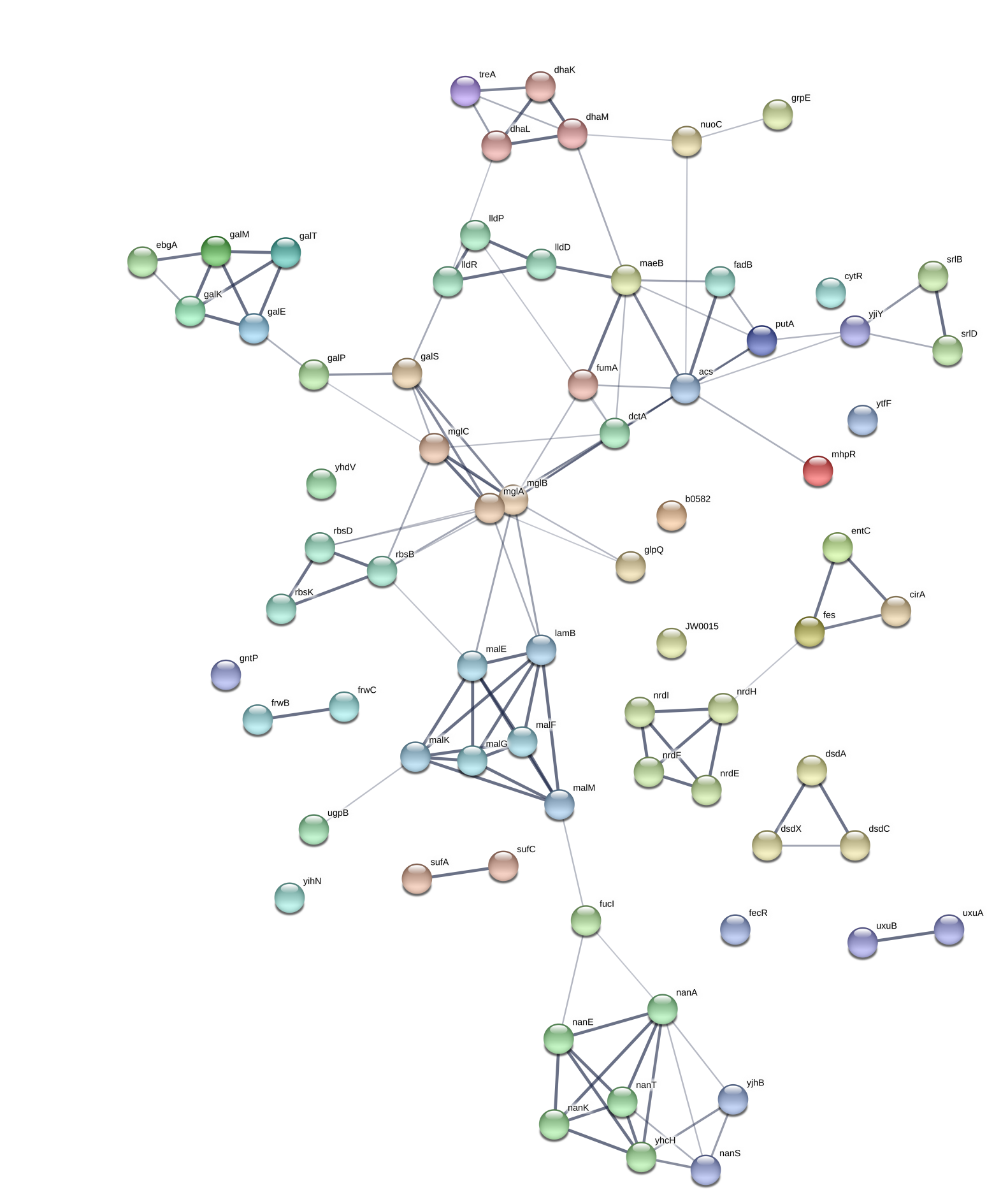
**

**Fig. S6. Growth phenotypes of MG1655 and the Stx2 lysogens in minimal medium supplemented with singe carbon sources.** The strains were grown in minimal medium supplemented with 0.2% glucose, maltose, L-lactate, galactose, sialic acid and ribose, or in the nutrient rich LB medium. The graphs represent the change in OD_595_ over time.

**
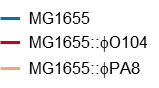
**

**Fig. S7. Bacterial size in growth experiments with minimal medium supplemented with single carbon sources.** The graph represents the length of 100 cells measured after 24h of incubation in minimal medium supplemented with 0.2% of the corresponding carbon sources. No significant difference (threshold 1.15 fold difference in the length) was found between the samples.

**
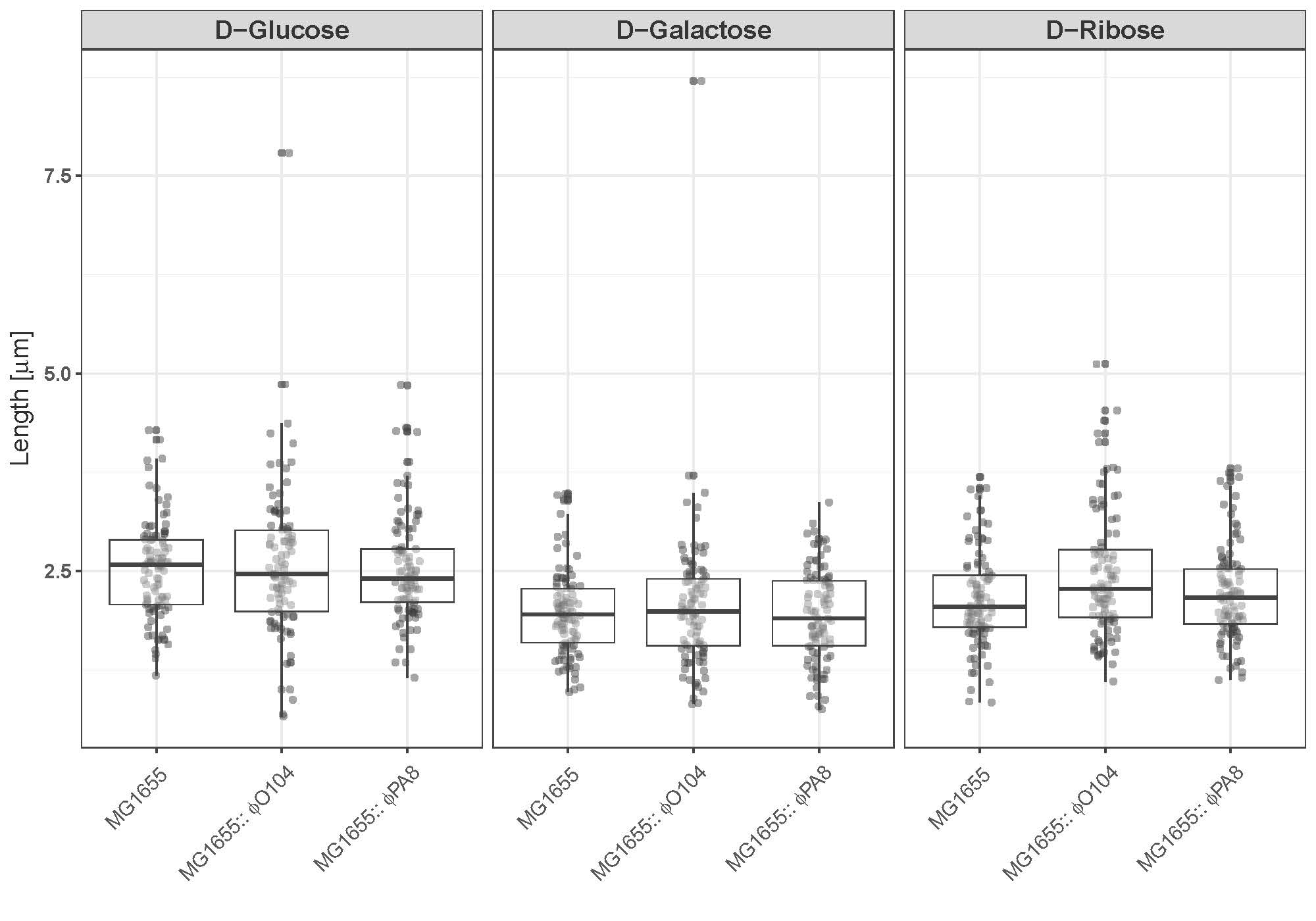
**

**Table S4. Semi-quantitative determination of *stx2* copy number in MG1655::φO104.** The *stx2* copy number was determined by quantitative PCR. The normalized increase in the *stx2* copies was calculated using the ΔΔCt method with *gapA* being the control gene and the exponential starter culture (OD = 0.4 in LB) set as control sample. The mean and standard deviation of three biological replicates are presented.

| **Growth medium^+^** | **Relative normalized increase in *stx2* copy numbers** |
| --- | --- |
| MM+ glucose | 1.20 ± 0.14 |
| MM+ maltose | 30.46 ± 3.36 |
| MM+ L-lactate | 28.86 ± 3.60 |
| MM+ galactose | 57.85 ± 5.57 |
| MM+ sialic acid | 27.27 ± 2.45 |
| MM+ ribose | 60.58 ± 9.57 |
| LB | 7808.58 ± 1388.72 |

**Fig. S8. Kinetic measurements of the respiration potential of the strains using BIOLOG PM1 MicroPlate^TM^ Carbon Sources.** Active cellular respiration (NADH production) leads to a reduction of a tetrazolium dye and formation of a blue color, which is recoded by OD measurements. The graphs represent the change in OD_595_ over time of three biological replicates per strain. **A. Lane A1-A12.**

**
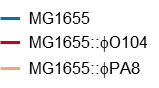
**

**B. Well B1-B12.**

**
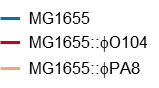
**

**C. Well C1-C12.**

**
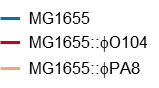
**

**D. Well D1-D12.**

**
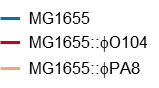
**

**E. E1-E12.**

**
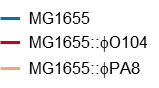
**

**F. F1-F12**

**
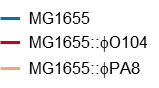
**

**G. G1-G12.**

**
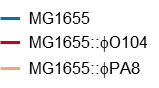
**

**H. H1- H12.**

**
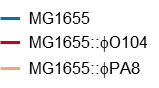
**

**Table S5. Statistical comparisons of the respiration potential of the strains using BIOLOG PM1 MicroPlate^TM^ Carbon Sources.** The statistical analysis (multiple comparison of means) was performed using the opm package and function opm_mcp with “Turkey”-type of comparison (all-against-all). The PM1 lane and substrate names, comparison strains and p value (adjusted for multiple comparisons) are given. The significant codes are given as follows: p < 0.001***, p < 0.01**, p < 0.05*.

| **Lane** | **Substrate** | **Comparison** | **p value** |
| --- | --- | --- | --- |
| A01 | - | MG1655::φPA8 vs. MG1655 | 1.000000 |
| A01 | - | MG1655::φO104 vs. MG1655 | 1.00000 |
| A01 | - | MG1655::φPA8 vs. MG1655::φO104 | 1.000000 |
| A02 | L-Arabinose | MG1655::φPA8 vs. MG1655 | < 1e-04*** |
| A02 | L-Arabinose | MG1655::φO104 vs. MG1655 | 0.000349*** |
| A02 | L-Arabinose | MG1655::φPA8 vs. MG1655::φO104 | 1.000000 |
| A03 | N-Acetyl-D-Glucosamie | MG1655::φPA8 vs. MG1655 | < 1e-04*** |
| A03 | N-Acetyl-D-Glucosamie | MG1655::φO104 vs. MG1655 | < 1e-04*** |
| A03 | N-Acetyl-D-Glucosamie | MG1655::φPA8 vs. MG1655::φO104 | 1.000000 |
| A04 | D-Saccharic Acid | MG1655::φPA8 vs. MG1655 | 0.001583** |
| A04 | D-Saccharic Acid | MG1655::φO104 vs. MG1655 | < 1e-04*** |
| A04 | D-Saccharic Acid | MG1655::φPA8 vs. MG1655::φO104 | 1.000000 |
| A05 | Succinic Acid | MG1655::φPA8 vs. MG1655 | 0.697644 |
| A05 | Succinic Acid | MG1655::φO104 vs. MG1655 | < 1e-04*** |
| A05 | Succinic Acid | MG1655::φPA8 vs. MG1655::φO104 | 0.000703*** |
| A06 | D-Galactose | MG1655::φPA8 vs. MG1655 | 1.000000 |
| A06 | D-Galactose | MG1655::φO104 vs. MG1655 | 1.000000 |
| A06 | D-Galactose | MG1655::φPA8 vs. MG1655::φO104 | 1.000000 |
| A07 | L-Aspartic Acid | MG1655::φPA8 vs. MG1655 | < 1e-04*** |
| A07 | L-Aspartic Acid | MG1655::φO104 vs. MG1655 | < 1e-04*** |
| A07 | L-Aspartic Acid | MG1655::φPA8 vs. MG1655::φO104 | < 1e-04*** |
| A08 | L-Proline | MG1655::φPA8 vs. MG1655 | < 1e-04*** |
| A08 | L-Proline | MG1655::φO104 vs. MG1655 | < 1e-04*** |
| A08 | L-Proline | MG1655::φPA8 vs. MG1655::φO104 | < 1e-04*** |
| A09 | D-Alanine | MG1655::φPA8 vs. MG1655 | < 1e-04*** |
| A09 | D-Alanine | MG1655::φO104 vs. MG1655 | < 1e-04*** |
| A09 | D-Alanine | MG1655::φPA8 vs. MG1655::φO104 | < 1e-04*** |
| A10 | D-Trehalose | MG1655::φPA8 vs. MG1655 | 0.999984 |
| A10 | D-Trehalose | MG1655::φO104 vs. MG1655 | 1.000000 |
| A10 | D-Trehalose | MG1655::φPA8 vs. MG1655::φO104 | 1.000000 |
| A11 | D-Mannose | MG1655::φPA8 vs. MG1655 | 0.000309*** |
| A11 | D-Mannose | MG1655::φO104 vs. MG1655 | < 1e-04*** |
| A11 | D-Mannose | MG1655::φPA8 vs. MG1655::φO104 | 1.000000 |
| A12 | Dulcitol | MG1655::φPA8 vs. MG1655 | 0.945468 |
| A12 | Dulcitol | MG1655::φO104 vs. MG1655 | 0.026613* |
| A12 | Dulcitol | MG1655::φPA8 vs. MG1655::φO104 | 1.000000 |
| B01 | D-Serine | MG1655::φPA8 vs. MG1655 | 0.136408 |
| **Lane** | **Substrate** | **Comparison** | **p value** |
| B01 | D-Serine | MG1655::φO104 vs. MG1655 | < 1e-04*** |
| B01 | D-Serine | MG1655::φPA8 vs. MG1655::φO104 | < 1e-04*** |
| B02 | D-Sorbitol | MG1655::φPA8 vs. MG1655 | 0.422235 |
| B02 | D-Sorbitol | MG1655::φO104 vs. MG1655 | 0.052062 |
| B02 | D-Sorbitol | MG1655::φPA8 vs. MG1655::φO104 | 1.000000 |
| B03 | Glycerol | MG1655::φPA8 vs. MG1655 | < 1e-04*** |
| B03 | Glycerol | MG1655::φO104 vs. MG1655 | < 1e-04*** |
| B03 | Glycerol | MG1655::φPA8 vs. MG1655::φO104 | 1.000000 |
| B04 | L-Fucose | MG1655::φO104 vs. MG1655 | 0.754228 |
| B04 | L-Fucose | MG1655::φPA8 vs. MG1655 | 0.046932 * |
| B04 | L-Fucose | MG1655::φPA8 vs. MG1655::φO104 | 1.000000 |
| B05 | D-Glucuronic Acid | MG1655::φPA8 vs. MG1655 | < 1e-04*** |
| B05 | D-Glucuronic Acid | MG1655::φO104 vs. MG1655 | < 1e-04*** |
| B05 | D-Glucuronic Acid | MG1655::φPA8 vs. MG1655::φO104 | 0.005382** |
| B06 | D-Gluconic Acid | MG1655::φPA8 vs. MG1655 | 0.047903 * |
| B06 | D-Gluconic Acid | MG1655::φO104 vs. MG1655 | < 1e-04*** |
| B06 | D-Gluconic Acid | MG1655::φPA8 vs. MG1655::φO104 | 0.248714 |
| B07 | D,L-α-Glycerol-Phosphate | MG1655::φPA8 vs. MG1655 | < 1e-04*** |
| B07 | D,L-α-Glycerol-Phosphate | MG1655::φO104 vs. MG1655 | < 1e-04*** |
| B07 | D,L-α-Glycerol-Phosphate | MG1655::φPA8 vs. MG1655::φO104 | 0.596599 |
| B08 | D-Xylose | MG1655::φPA8 vs. MG1655 | < 1e-04*** |
| B08 | D-Xylose | MG1655::φO104 vs. MG1655 | 0.000102*** |
| B08 | D-Xylose | MG1655::φPA8 vs. MG1655::φO104 | 1.000000 |
| B09 | L-Lactic Acid | MG1655::φPA8 vs. MG1655 | 9 0.0054 ** |
| B09 | L-Lactic Acid | MG1655::φO104 vs. MG1655 | < 1e-04*** |
| B09 | L-Lactic Acid | MG1655::φPA8 vs. MG1655::φO104 | < 1e-04*** |
| B10 | Formic Acid | MG1655::φPA8 vs. MG1655 | 1.000000 |
| B10 | Formic Acid | MG1655::φO104 vs. MG1655 | 1.000000 |
| B10 | Formic Acid | MG1655::φPA8 vs. MG1655::φO104 | 1.000000 |
| B11 | D-Mannitol | MG1655::φO104 vs. MG1655 | 0.010560* |
| B11 | D-Mannitol | MG1655::φPA8 vs. MG1655 | 0.000356*** |
| B11 | D-Mannitol | MG1655::φPA8 vs. MG1655::φO104 | 1.000000 |
| B12 | L-Glutamic Acid | MG1655::φPA8 vs. MG1655 | < 1e-04*** |
| B12 | L-Glutamic Acid | MG1655::φO104 vs. MG1655 | < 1e-04*** |
| B12 | L-Glutamic Acid | MG1655::φPA8 vs. MG1655::φO104 | 1.000000 |
| C01 | D-Glucose-6-Phosphate | MG1655::φPA8 vs. MG1655 | 0.999964 |
| C01 | D-Glucose-6-Phosphate | MG1655::φO104 vs. MG1655 | < 1e-04*** |
| C01 | D-Glucose-6-Phosphate | MG1655::φPA8 vs. MG1655::φO104 | 0.239006 |
| C02 | D-Galactonic Acid-γ-Lactone | MG1655::φPA8 vs. MG1655 | 1.000000 |
| C02 | D-Galactonic Acid-γ-Lactone | MG1655::φO104 vs. MG1655 | 1.000000 |
| C02 | D-Galactonic Acid-γ-Lactone | MG1655::φPA8 vs. MG1655::φO104 | 1.000000 |
| C03 | D,L-Malic Acid | MG1655::φPA8 vs. MG1655 | < 1e-04*** |
| C03 | D,L-Malic Acid | MG1655::φO104 vs. MG1655 | < 1e-04*** |
| C03 | D,L-Malic Acid | MG1655::φPA8 vs. MG1655::φO104 | < 1e-04*** |
| C04 | D-Ribose | MG1655::φPA8 vs. MG1655 | 1.000000 |
| C04 | D-Ribose | MG1655::φO104 vs. MG1655 | < 1e-04*** |
| **Lane** | **Substrate** | **Comparison** | **p value** |
| C04 | D-Ribose | MG1655::φPA8 vs. MG1655::φO104 | < 1e-04*** |
| C05 | Tween 20 | MG1655::φPA8 vs. MG1655 | 0.469430 |
| C05 | Tween 20 | MG1655::φO104 vs. MG1655 | 0.508362 |
| C05 | Tween 20 | MG1655::φPA8 vs. MG1655::φO104 | 1.000000 |
| C06 | L-Rhamnose | MG1655::φPA8 vs. MG1655 | 0.001541** |
| C06 | L-Rhamnose | MG1655::φO104 vs. MG1655 | < 1e-04*** |
| C06 | L-Rhamnose | MG1655::φPA8 vs. MG1655::φO104 | 0.154585 |
| C07 | D-Fructose | MG1655::φPA8 vs. MG1655 | < 1e-04*** |
| C07 | D-Fructose | MG1655::φO104 vs. MG1655 | < 1e-04*** |
| C07 | D-Fructose | MG1655::φPA8 vs. MG1655::φO104 | 1.000000 |
| C08 | Acetic Acid | MG1655::φPA8 vs. MG1655 | < 1e-04*** |
| C08 | Acetic Acid | MG1655::φO104 vs. MG1655 | < 1e-04*** |
| C08 | Acetic Acid | MG1655::φPA8 vs. MG1655::φO104 | < 1e-04*** |
| C09 | α-D-Glucose | MG1655::φPA8 vs. MG1655 | 0.980444 |
| C09 | α-D-Glucose | MG1655::φO104 vs. MG1655 | 0.256873 |
| C09 | α-D-Glucose | MG1655::φPA8 vs. MG1655::φO104 | 1.000000 |
| C10 | Maltose | MG1655::φPA8 vs. MG1655 | 0.011135 * |
| C10 | Maltose | MG1655::φO104 vs. MG1655 | 0.000259*** |
| C10 | Maltose | MG1655::φPA8 vs. MG1655::φO104 | 1.000000 |
| C11 | D-Melibiose | MG1655::φPA8 vs. MG1655 | 0.999817 |
| C11 | D-Melibiose | MG1655::φO104 vs. MG1655 | 0.622540 |
| C11 | D-Melibiose | MG1655::φPA8 vs. MG1655::φO104 | 1.000000 |
| C12 | Thymidine | MG1655::φPA8 vs. MG1655 | < 1e-04*** |
| C12 | Thymidine | MG1655::φO104 vs. MG1655 | < 1e-04*** |
| C12 | Thymidine | MG1655::φPA8 vs. MG1655::φO104 | < 1e-04*** |
| D01 | L-Asparagine | MG1655::φPA8 vs. MG1655 | < 1e-04*** |
| D01 | L-Asparagine | MG1655::φO104 vs. MG1655 | < 1e-04*** |
| D01 | L-Asparagine | MG1655::φPA8 vs. MG1655::φO104 | < 1e-04*** |
| D02 | D-Aspartic Acid | MG1655::φPA8 vs. MG1655 | 1.000000 |
| D02 | D-Aspartic Acid | MG1655::φO104 vs. MG1655 | 1.000000 |
| D02 | D-Aspartic Acid | MG1655::φPA8 vs. MG1655::φO104 | 1.000000 |
| D03 | D-Glucosaminic Acid | MG1655::φPA8 vs. MG1655 | 1.000000 |
| D03 | D-Glucosaminic Acid | MG1655::φO104 vs. MG1655 | 1.000000 |
| D03 | D-Glucosaminic Acid | MG1655::φPA8 vs. MG1655::φO104 | 1.000000 |
| D04 | 1,2-Propanediol | MG1655::φPA8 vs. MG1655 | 1.000000 |
| D04 | 1,2-Propanediol | MG1655::φO104 vs. MG1655 | 1.000000 |
| D04 | 1,2-Propanediol | MG1655::φPA8 vs. MG1655::φO104 | 1.000000 |
| D05 | Tween 40 | MG1655::φPA8 vs. MG1655 | 1.000000 |
| D05 | Tween 40 | MG1655::φO104 vs. MG1655 | 1.000000 |
| D05 | Tween 40 | MG1655::φPA8 vs. MG1655::φO104 | 1.000000 |
| D06 | α-Keto-Glutaric Acid | MG1655::φPA8 vs. MG1655 | < 1e-04*** |
| D06 | α-Keto-Glutaric Acid | MG1655::φO104 vs. MG1655 | < 1e-04*** |
| D06 | α-Keto-Glutaric Acid | MG1655::φPA8 vs. MG1655::φO104 | < 1e-04*** |
| D07 | α-Keto-Butyric Acid | MG1655::φPA8 vs. MG1655 | 0.000471*** |
| D07 | α-Keto-Butyric Acid | MG1655::φO104 vs. MG1655 | < 1e-04*** |
| D07 | α-Keto-Butyric Acid | MG1655::φPA8 vs. MG1655::φO104 | 0.253194 |
| **Lane** | **Substrate** | **Comparison** | **p value** |
| D08 | α-Methyl-D-Galactoside | MG1655::φPA8 vs. MG1655 | < 1e-04*** |
| D08 | α-Methyl-D-Galactoside | MG1655::φO104 vs. MG1655 | < 1e-04*** |
| D08 | α-Methyl-D-Galactoside | MG1655::φPA8 vs. MG1655::φO104 | < 1e-04*** |
| D09 | α-D-Lactose | MG1655::φPA8 vs. MG1655 | 0.815684 |
| D09 | α-D-Lactose | MG1655::φO104 vs. MG1655 | 0.009567** |
| D09 | α-D-Lactose | MG1655::φPA8 vs. MG1655::φO104 | 1.000000 |
| D10 | Lactulose | MG1655::φPA8 vs. MG1655 | < 1e-04*** |
| D10 | Lactulose | MG1655::φO104 vs. MG1655 | < 1e-04*** |
| D10 | Lactulose | MG1655::φPA8 vs. MG1655::φO104 | 1.000000 |
| D11 | Sucrose | MG1655::φPA8 vs. MG1655 | 1.000000 |
| D11 | Sucrose | MG1655::φO104 vs. MG1655 | 1.000000 |
| D11 | Sucrose | MG1655::φPA8 vs. MG1655::φO104 | 1.000000 |
| D12 | Uridine | MG1655::φPA8 vs. MG1655 | 0.005832** |
| D12 | Uridine | MG1655::φO104 vs. MG1655 | < 1e-04*** |
| D12 | Uridine | MG1655::φPA8 vs. MG1655::φO104 | < 1e-04*** |
| E01 | L-Glutamine | MG1655::φPA8 vs. MG1655 | < 1e-04*** |
| E01 | L-Glutamine | MG1655::φO104 vs. MG1655 | < 1e-04*** |
| E01 | L-Glutamine | MG1655::φPA8 vs. MG1655::φO104 | 0.985794 |
| E02 | m-Tartaric Acid | MG1655::φPA8 vs. MG1655 | < 1e-04*** |
| E02 | m-Tartaric Acid | MG1655::φO104 vs. MG1655 | < 1e-04*** |
| E02 | m-Tartaric Acid | MG1655::φPA8 vs. MG1655::φO104 | 0.999919 |
| E03 | D-Glucose-1-Phosphate | MG1655::φPA8 vs. MG1655 | 0.884010 |
| E03 | D-Glucose-1-Phosphate | MG1655::φO104 vs. MG1655 | < 1e-04*** |
| E03 | D-Glucose-1-Phosphate | MG1655::φPA8 vs. MG1655::φO104 | < 1e-04*** |
| E04 | D-Fructose-6-Phosphate | MG1655::φPA8 vs. MG1655 | 0.036938 * |
| E04 | D-Fructose-6-Phosphate | MG1655::φO104 vs. MG1655 | < 1e-04*** |
| E04 | D-Fructose-6-Phosphate | MG1655::φPA8 vs. MG1655::φO104 | 0.029742 * |
| E05 | Tween 80 | MG1655::φPA8 vs. MG1655 | 1.000000 |
| E05 | Tween 80 | MG1655::φO104 vs. MG1655 | 1.000000 |
| E05 | Tween 80 | MG1655::φPA8 vs. MG1655::φO104 | 1.000000 |
| E06 | α-Hydroxy Glutaric Acid-γ-Lactone | MG1655::φPA8 vs. MG1655 | 1.000000 |
| E06 | α-Hydroxy Glutaric Acid-γ-Lactone | MG1655::φO104 vs. MG1655 | 1.000000 |
| E06 | α-Hydroxy Glutaric Acid- γ-Lactone | MG1655::φPA8 vs. MG1655::φO104 | 1.000000 |
| E07 | α-Hydroxy Butyric Acid | MG1655::φO104 vs. MG1655 | < 1e-04*** |
| E07 | α-Hydroxy Butyric Acid | MG1655::φPA8 vs. MG1655 | 0.076482 |
| E07 | α-Hydroxy Butyric Acid | MG1655::φPA8 vs. MG1655::φO104 | 0.052530 |
| E08 | β-Methyl-D-Glucoside | MG1655::φPA8 vs. MG1655 | < 1e-04*** |
| E08 | β-Methyl-D-Glucoside | MG1655::φO104 vs. MG1655 | < 1e-04*** |
| E08 | β-Methyl-D-Glucoside | MG1655::φPA8 vs. MG1655::φO104 | 1.000000 |
| E09 | Adonitol | MG1655::φPA8 vs. MG1655 | 1.000000 |
| E09 | Adonitol | MG1655::φO104 vs. MG1655 | 1.000000 |
| E09 | Adonitol | MG1655::φPA8 vs. MG1655::φO104 | 1.000000 |
| E10 | Maltotriose | MG1655::φPA8 vs. MG1655 | 1.000000 |
| E10 | Maltotriose | MG1655::φO104 vs. MG1655 | 1.000000 |
| E10 | Maltotriose | MG1655::φPA8 vs. MG1655::φO104 | 1.000000 |
| E11 | 2-Deoxy Adenosine | MG1655::φPA8 vs. MG1655 | < 1e-04*** |
| **Lane** | **Substrate** | **Comparison** | **p value** |
| E11 | 2-Deoxy Adenosine | MG1655::φO104 vs. MG1655 | < 1e-04*** |
| E11 | 2-Deoxy Adenosine | MG1655::φPA8 vs. MG1655::φO104 | < 1e-04*** |
| E12 | Adenosine | MG1655::φPA8 vs. MG1655 | < 1e-04*** |
| E12 | Adenosine | MG1655::φO104 vs. MG1655 | < 1e-04*** |
| E12 | Adenosine | MG1655::φPA8 vs. MG1655::φO104 | 0.001297** |
| F01 | Glycyl-L-Aspartic Acid | MG1655::φPA8 vs. MG1655 | < 1e-04*** |
| F01 | Glycyl-L-Aspartic Acid | MG1655::φO104 vs. MG1655 | < 1e-04*** |
| F01 | Glycyl-L-Aspartic Acid | MG1655::φPA8 vs. MG1655::φO104 | < 1e-04*** |
| F02 | Citric Acid | MG1655::φPA8 vs. MG1655 | 1.000000 |
| F02 | Citric Acid | MG1655::φO104 vs. MG1655 | 1.000000 |
| F02 | Citric Acid | MG1655::φPA8 vs. MG1655::φO104 | 1.000000 |
| F03 | m-Inositol | MG1655::φPA8 vs. MG1655 | 1.000000 |
| F03 | m-Inositol | MG1655::φO104 vs. MG1655 | 1.000000 |
| F03 | m-Inositol | MG1655::φPA8 vs. MG1655::φO104 | 1.000000 |
| F04 | D-Threonine | MG1655::φPA8 vs. MG1655 | 1.000000 |
| F04 | D-Threonine | MG1655::φO104 vs. MG1655 | 1.000000 |
| F04 | D-Threonine | MG1655::φPA8 vs. MG1655::φO104 | 1.000000 |
| F05 | Fumaric Acid | MG1655::φO104 vs. MG1655 | 0.002186** |
| F05 | Fumaric Acid | MG1655::φPA8 vs. MG1655 | 0.065601 |
| F05 | Fumaric Acid | MG1655::φPA8 vs. MG1655::φO104 | 1.000000 |
| F06 | Bromo Succinic Acid | MG1655::φPA8 vs. MG1655 | < 1e-04*** |
| F06 | Bromo Succinic Acid | MG1655::φO104 vs. MG1655 | < 1e-04*** |
| F06 | Bromo Succinic Acid | MG1655::φPA8 vs. MG1655::φO104 | 0.010230* |
| F07 | Propionic Acid | MG1655::φPA8 vs. MG1655 | < 1e-04*** |
| F07 | Propionic Acid | MG1655::φO104 vs. MG1655 | < 1e-04*** |
| F07 | Propionic Acid | MG1655::φPA8 vs. MG1655::φO104 | 0.168996 |
| F08 | Mucic Acid | MG1655::φPA8 vs. MG1655 | 0.017000 * |
| F08 | Mucic Acid | MG1655::φO104 vs. MG1655 | < 1e-04*** |
| F08 | Mucic Acid | MG1655::φPA8 vs. MG1655::φO104 | < 1e-04*** |
| F09 | Glycolic Acid | MG1655::φPA8 vs. MG1655 | < 1e-04*** |
| F09 | Glycolic Acid | MG1655::φO104 vs. MG1655 | < 1e-04*** |
| F09 | Glycolic Acid | MG1655::φPA8 vs. MG1655::φO104 | < 1e-04*** |
| F10 | Glyoxylic Acid | MG1655::φPA8 vs. MG1655 | < 1e-04*** |
| F10 | Glyoxylic Acid | MG1655::φO104 vs. MG1655 | < 1e-04*** |
| F10 | Glyoxylic Acid | MG1655::φPA8 vs. MG1655::φO104 | 1.000000 |
| F11 | D-Cellobiose | MG1655::φPA8 vs. MG1655 | 1.000000 |
| F11 | D-Cellobiose | MG1655::φO104 vs. MG1655 | 1.000000 |
| F11 | D-Cellobiose | MG1655::φPA8 vs. MG1655::φO104 | 1.000000 |
| F12 | Inosine | MG1655::φPA8 vs. MG1655 | < 1e-04*** |
| F12 | Inosine | MG1655::φO104 vs. MG1655 | < 1e-04*** |
| F12 | Inosine | MG1655::φPA8 vs. MG1655::φO104 | 0.005487 ** |
| G01 | Glycyl-L-Glutamic Acid | MG1655::φPA8 vs. MG1655 | < 1e-04*** |
| G01 | Glycyl-L-Glutamic Acid | MG1655::φO104 vs. MG1655 | < 1e-04*** |
| G01 | Glycyl-L-Glutamic Acid | MG1655::φPA8 vs. MG1655::φO104 | 1.000000 |
| G02 | Tricarballylic Acid | MG1655::φPA8 vs. MG1655 | 1.000000 |
| G02 | Tricarballylic Acid | MG1655::φO104 vs. MG1655 | 1.000000 |
| **Lane** | **Substrate** | **Comparison** | **p value** |
| G02 | Tricarballylic Acid | MG1655::φPA8 vs. MG1655::φO104 | 1.000000 |
| G03 | L-Serine | MG1655::φPA8 vs. MG1655 | < 1e-04*** |
| G03 | L-Serine | MG1655::φO104 vs. MG1655 | < 1e-04*** |
| G03 | L-Serine | MG1655::φPA8 vs. MG1655::φO104 | < 1e-04*** |
| G04 | L-Threonine | MG1655::φPA8 vs. MG1655 | < 1e-04*** |
| G04 | L-Threonine | MG1655::φO104 vs. MG1655 | < 1e-04*** |
| G04 | L-Threonine | MG1655::φPA8 vs. MG1655::φO104 | 1.000000 |
| G05 | L-Alanine | MG1655::φPA8 vs. MG1655 | < 1e-04*** |
| G05 | L-Alanine | MG1655::φO104 vs. MG1655 | < 1e-04*** |
| G05 | L-Alanine | MG1655::φPA8 vs. MG1655::φO104 | < 1e-04*** |
| G06 | L-Alanyl-Glycine | MG1655::φPA8 vs. MG1655 | 0.000541*** |
| G06 | L-Alanyl-Glycine | MG1655::φO104 vs. MG1655 | < 1e-04*** |
| G06 | L-Alanyl-Glycine | MG1655::φPA8 vs. MG1655::φO104 | < 1e-04*** |
| G07 | Acetoacetic Acid | MG1655::φPA8 vs. MG1655 | 1.000000 |
| G07 | Acetoacetic Acid | MG1655::φO104 vs. MG1655 | 1.000000 |
| G07 | Acetoacetic Acid | MG1655::φPA8 vs. MG1655::φO104 | 1.000000 |
| G08 | N-Acetyl-β-D-Mannosamine | MG1655::φPA8 vs. MG1655 | < 1e-04*** |
| G08 | N-Acetyl-β-D-Mannosamine | MG1655::φO104 vs. MG1655 | < 1e-04*** |
| G08 | N-Acetyl-β-D-Mannosamine | MG1655::φPA8 vs. MG1655::φO104 | 1.000000 |
| G09 | Mono Methyl Succinate | MG1655::φPA8 vs. MG1655 | 1.000000 |
| G09 | Mono Methyl Succinate | MG1655::φO104 vs. MG1655 | 1.000000 |
| G09 | Mono Methyl Succinate | MG1655::φPA8 vs. MG1655::φO104 | 1.000000 |
| G10 | Methyl Pyruvate | MG1655::φPA8 vs. MG1655 | < 1e-04*** |
| G10 | Methyl Pyruvate | MG1655::φO104 vs. MG1655 | < 1e-04*** |
| G10 | Methyl Pyruvate | MG1655::φPA8 vs. MG1655::φO104 | 1.000000 |
| G11 | D-Malic Acid | MG1655::φPA8 vs. MG1655 | 0.000100*** |
| G11 | D-Malic Acid | MG1655::φO104 vs. MG1655 | < 1e-04*** |
| G11 | D-Malic Acid | MG1655::φPA8 vs. MG1655::φO104 | < 1e-04*** |
| G12 | L-Malic Acid | MG1655::φPA8 vs. MG1655 | 0.009330** |
| G12 | L-Malic Acid | MG1655::φO104 vs. MG1655 | < 1e-04*** |
| G12 | L-Malic Acid | MG1655::φPA8 vs. MG1655::φO104 | < 1e-04*** |
| H01 | Glycyl-L-Proline | MG1655::φPA8 vs. MG1655 | < 1e-04*** |
| H01 | Glycyl-L-Proline | MG1655::φO104 vs. MG1655 | < 1e-04*** |
| H01 | Glycyl-L-Proline | MG1655::φPA8 vs. MG1655::φO104 | < 1e-04*** |
| H02 | p-Hydroxy Phenyl Acetic Acid | MG1655::φPA8 vs. MG1655 | 1.000000 |
| H02 | p-Hydroxy Phenyl Acetic Acid | MG1655::φO104 vs. MG1655 | 1.000000 |
| H02 | p-Hydroxy Phenyl Acetic Acid | MG1655::φPA8 vs. MG1655::φO104 | 1.000000 |
| H03 | m-Hydroxy Phenyl Acetic Acid | MG1655::φPA8 vs. MG1655 | 1.000000 |
| H03 | m-Hydroxy Phenyl Acetic Acid | MG1655::φO104 vs. MG1655 | 1.000000 |
| H03 | m-Hydroxy Phenyl Acetic Acid | MG1655::φPA8 vs. MG1655::φO104 | 1.000000 |
| H04 | Tyramine | MG1655::φPA8 vs. MG1655 | 1.000000 |
| H04 | Tyramine | MG1655::φO104 vs. MG1655 | 1.000000 |
| H04 | Tyramine | MG1655::φPA8 vs. MG1655::φO104 | 1.000000 |
| H05 | D-Psicose | MG1655::φPA8 vs. MG1655 | 1.000000 |
| H05 | D-Psicose | MG1655::φO104 vs. MG1655 | 0.805299 |
| H05 | D-Psicose | MG1655::φPA8 vs. MG1655::φO104 | 1.000000 |
| **Lane** | **Substrate** | **Comparison** | **p value** |
| H06 | L-Lyxose | MG1655::φPA8 vs. MG1655 | 0.486429 |
| H06 | L-Lyxose | MG1655::φO104 vs. MG1655 | 0.375687 |
| H06 | L-Lyxose | MG1655::φPA8 vs. MG1655::φO104 | 1.000000 |
| H07 | Glucuronamide | MG1655::φPA8 vs. MG1655 | 1.000000 |
| H07 | Glucuronamide | MG1655::φO104 vs. MG1655 | 1.000000 |
| H07 | Glucuronamide | MG1655::φPA8 vs. MG1655::φO104 | 1.000000 |
| H08 | Pyruvic Acid | MG1655::φPA8 vs. MG1655 | < 1e-04*** |
| H08 | Pyruvic Acid | MG1655::φO104 vs. MG1655 | < 1e-04*** |
| H08 | Pyruvic Acid | MG1655::φPA8 vs. MG1655::φO104 | 0.600691 |
| H09 | L-Galactonic Acid-γ-Lactone | MG1655::φPA8 vs. MG1655 | 0.999998 |
| H09 | L-Galactonic Acid-γ-Lactone | MG1655::φO104 vs. MG1655 | 0.626588 |
| H09 | L-Galactonic Acid-γ-Lactone | MG1655::φPA8 vs. MG1655::φO104 | 1.000000 |
| H10 | D-Galacturonic Acid | MG1655::φPA8 vs. MG1655 | 0.024029 * |
| H10 | D-Galacturonic Acid | MG1655::φO104 vs. MG1655 | < 1e-04*** |
| H10 | D-Galacturonic Acid | MG1655::φPA8 vs. MG1655::φO104 | 0.908449 |
| H11 | Phenylethylamine | MG1655::φPA8 vs. MG1655 | 1.000000 |
| H11 | Phenylethylamine | MG1655::φO104 vs. MG1655 | 1.000000 |
| H11 | Phenylethylamine | MG1655::φPA8 vs. MG1655::φO104 | 1.000000 |
| H12 | 2-Aminoethanol | MG1655::φPA8 vs. MG1655 | 1.000000 |
| H12 | 2-Aminoethanol | MG1655::φO104 vs. MG1655 | 1.000000 |
| H12 | 2-Aminoethanol | MG1655::φPA8 vs. MG1655::φO104 | 1.000000 |

**Table S6. Normalized counts of sequencing reads mapped to A. φO104-encoded genes and B. φPA8-encoded genes.** The protein_ID of each gene is given. The Start and End coordinates of φO104- encoded genes are based on the sequence in NC_018658. The rpkm (reads per kilobase of transcript) normalized number of reads is shown. The *cI* and *cro* genes are marked in bold.

**A.**

| **Protein_ID** | **Start** | **End** | **Strand** | **RPMK normalized countings** | | |
| --- | --- | --- | --- | --- | --- | --- |
|  |  |  |  | **1** | **2** | **3** |
| YP_006779868.1 | 3256637 | 3265018 | - | 14.36182708 | 15.45767582 | 23.26080256 |
| YP_006779869.1 | 3265088 | 3266353 | - | 10.60999133 | 12.91549049 | 20.2512129 |
| YP_006779870.1 | 3266364 | 3266615 | - | 6.862307623 | 6.065196256 | 14.32185807 |
| YP_006779871.1 | 3266625 | 3267071 | - | 8.187213902 | 9.794884894 | 19.07872279 |
| YP_006779872.1 | 3267074 | 3267730 | - | 10.01837158 | 12.4315752 | 19.26729521 |
| YP_006779873.1 | 3267824 | 3268225 | - | 76.96455533 | 82.18001457 | 100.3527607 |
| YP_006779874.1 | 3268282 | 3268422 | - | 1107.327221 | 1334.891625 | 1646.614156 |
| YP_006779875.1 | 3268655 | 3269389 | - | 31.16992353 | 26.60021787 | 32.68111613 |
| YP_006779876.1 | 3269480 | 3270097 | - | 17.59192611 | 16.90010996 | 26.49620992 |
| YP_006779877.1 | 3270103 | 3270381 | - | 28.92499557 | 31.84228034 | 50.64154259 |
| YP_006779878.1 | 3270396 | 3271664 | - | 7.88058066 | 9.823682234 | 15.02083397 |
| YP_006779879.1 | 3271661 | 3273352 | - | 23.34067422 | 31.32474919 | 36.19062433 |
| YP_006779880.1 | 3273520 | 3274032 | - | 5.853446676 | 6.020860026 | 9.588851565 |
| YP_006779881.1 | 3274119 | 3276311 | - | 15.31875422 | 18.74527311 | 25.83202563 |
| YP_006779882.1 | 3276308 | 3276958 | - | 30.04383142 | 41.74713713 | 50.65523135 |
| YP_006779883.1 | 3276958 | 3277521 | - | 13.38166964 | 15.49770559 | 23.10796249 |
| YP_006779884.1 | 3277505 | 3277966 | - | 29.56740578 | 34.84041713 | 49.67804103 |
| YP_006779885.1 | 3278016 | 3278405 | - | 34.81976626 | 42.29308005 | 59.12356794 |
| YP_006779886.1 | 3278461 | 3279675 | - | 64.85363409 | 74.8228069 | 102.1728034 |
| YP_006779887.1 | 3279699 | 3280706 | - | 75.3257953 | 88.21385701 | 120.7677421 |
| YP_006779888.1 | 3280864 | 3283008 | - | 20.32376916 | 23.30647093 | 31.73198503 |
| YP_006779889.1 | 3283008 | 3284714 | - | 11.00235433 | 14.02776498 | 17.3294203 |
| YP_006779890.1 | 3284695 | 3285501 | - | 15.89715527 | 21.54384767 | 27.18136038 |
| YP_006779891.1 | 3285794 | 3286345 | - | 52.84462574 | 53.18583782 | 78.55573747 |
| YP_006779892.1 | 3286548 | 3286976 | - | 22.12366016 | 27.46303899 | 42.74968918 |
| YP_006779893.1 | 3286988 | 3287122 | - | 17.77461799 | 16.51081203 | 31.88293145 |
| YP_006779894.1 | 3287137 | 3287706 | - | 20.06112059 | 21.2281869 | 32.45617801 |
| YP_006779895.1 | 3287980 | 3288513 | - | 18.8780702 | 19.61818396 | 29.66287458 |
| YP_006779896.1 | 3288518 | 3288733 | - | 17.31535761 | 16.87935694 | 27.29109622 |
| YP_006779897.1 | 3288811 | 3289056 | - | 85.82750033 | 85.49522832 | 103.4589292 |
| YP_006779898.1 | 3289097 | 3289276 | - | 87.13534795 | 99.77247841 | 118.0757632 |
| YP_006779899.1 | 3289413 | 3291350 | - | 32.10941376 | 36.87000882 | 56.11685318 |
| YP_006779900.1 | 3291836 | 3292105 | - | 79.63823254 | 86.5048616 | 113.3725358 |
| YP_006779901.1 | 3292117 | 3293076 | - | 48.25957733 | 52.68902326 | 70.90115405 |
| YP_006779902.1 | 3293459 | 3293557 | - | 52.80930179 | 54.67866322 | 81.01253051 |
| YP_006779903.1 | 3293860 | 3294294 | - | 5.916883044 | 7.612867025 | 12.90613417 |
| YP_006779904.1 | 3294287 | 3294481 | - | 12.3742506 | 14.69643708 | 19.193738 |
| **Protein_ID** | **Start** | **End** | **Strand** | **RPMK normalized countings** | | |
|  |  |  |  | **1** | **2** | **3** |
| YP_006779905.1 | 3294478 | 3295041 | - | 15.73475187 | 16.71155139 | 20.00320035 |
| YP_006779906.1 | 3295049 | 3295498 | - | 21.0018531 | 24.6246968 | 26.08063399 |
| YP_006779907.1 | 3295498 | 3296469 | - | 19.01862058 | 26.69903136 | 29.539569 |
| YP_006779908.1 | 3296459 | 3297979 | - | 34.63732539 | 44.83250714 | 51.52604665 |
| YP_006779909.1 | 3297973 | 3298350 | - | 50.21719687 | 56.22942362 | 75.21627684 |
| YP_006779910.1 | 3298558 | 3298851 | - | 54.67047734 | 61.19349794 | 93.25120923 |
| **YP_006779911.1 (Cro)** | **3298882** | **3299085** | **-** | **45.6704878** | **47.4512413** | **72.0118001** |
| **YP_006779912.1 (CI)** | **3299181** | **3299894** | **+** | **147.1781928** | **146.9472181** | **202.0973306** |
| YP_006779913.1 | 3299989 | 3301458 | + | 64.05428404 | 69.3923436 | 113.0835727 |
| YP_006779914.1 | 3301455 | 3302408 | + | 79.12580944 | 81.32464996 | 104.2603244 |
| YP_006779915.1 | 3303037 | 3303810 | + | 145.95878 | 99.62711744 | 107.7309655 |
| YP_006779916.1 | 3304267 | 3304740 | + | 87.82353149 | 72.19374953 | 89.22658582 |
| YP_006779917.1 | 3304811 | 3305023 | + | 273.1436693 | 243.974655 | 283.7834713 |
| YP_006779918.1 | 3305095 | 3305316 | + | 484.6488584 | 471.4665563 | 530.1065882 |
| YP_006779919.1 | 3305337 | 3305618 | + | 171.9889192 | 186.5370466 | 196.9983428 |
| YP_006779920.1 | 3305635 | 3306585 | + | 57.06121306 | 66.96588926 | 70.32117652 |
| YP_006779921.1 | 3306582 | 3307271 | + | 63.7438298 | 79.44418205 | 85.08429074 |
| YP_006779922.1 | 3307271 | 3307858 | + | 331.9213843 | 344.6060391 | 402.9443402 |
| YP_006779923.1 | 3307933 | 3308280 | + | 601.9349899 | 576.8210784 | 685.1774445 |
| YP_006779924.1 | 3308344 | 3309165 | + | 418.3899217 | 421.5034701 | 483.9951716 |
| YP_006779925.1 | 3309242 | 3309637 | + | 429.2109855 | 460.3460984 | 539.509698 |
| YP_006779926.1 | 3309788 | 3310513 | + | 105.2123782 | 118.1146845 | 137.3530631 |
| YP_006779927.1 | 3310510 | 3310917 | + | 58.68164836 | 73.44016129 | 78.76085871 |
| YP_006779928.1 | 3310919 | 3311110 | + | 86.99570797 | 103.9849468 | 107.3542611 |
| YP_006779929.1 | 3311113 | 3312009 | + | 57.47749734 | 69.25784722 | 75.01651947 |
| YP_006779930.1 | 3312002 | 3312100 | + | 68.24586693 | 84.91274759 | 92.08424301 |
| YP_006779931.1 | 3312365 | 3313003 | + | 94.34855059 | 87.03888837 | 99.17569199 |
| YP_006779932.1 | 3313059 | 3313490 | + | 29.94501808 | 32.72678813 | 38.67785745 |
| YP_006779933.1 | 3313487 | 3314113 | + | 38.20656773 | 48.6521604 | 57.98791658 |
| YP_006779934.1 | 3314073 | 3314285 | + | 96.67020185 | 98.66622079 | 127.8343501 |
| YP_006779935.1 | 3314321 | 3314674 | + | 111.6362476 | 114.2364295 | 149.9830289 |
| YP_006779936.1 | 3315039 | 3315290 | + | 227.3604867 | 199.2669687 | 261.4004318 |
| YP_006779937.1 | 3315321 | 3315620 | + | 81.77317269 | 83.53291544 | 101.8570546 |
| YP_006779938.1 | 3315673 | 3316983 | + | 84.5432209 | 96.5712484 | 122.3326287 |

**B.**

| **Protein_ID** | **Start** | **End** | **Strand** | **RPMK normalized countings** | | |
| --- | --- | --- | --- | --- | --- | --- |
|  |  |  |  | **1** | **2** | **3** |
| AKI86166.1 | 220 | 1389 | + | 101.066079 | 171.91425 | 162.208726 |
| AKI86167.1 | 1373 | 1540 | - | 186.614254 | 226.162967 | 218.866033 |
| AKI86168.1 | 1634 | 2020 | - | 122.142383 | 147.742878 | 140.909148 |
| AKI86169.1 | 2056 | 2268 | - | 73.8568783 | 99.6479087 | 100.024603 |
| AKI86170.1 | 2228 | 2854 | - | 42.5699933 | 53.326847 | 56.1618706 |
| AKI86171.1 | 2851 | 3282 | - | 19.0275533 | 29.7824566 | 30.8097006 |
| AKI86172.1 | 3338 | 4015 | - | 125.278758 | 85.4519193 | 95.0124324 |
| AKI86173.1 | 4340 | 4597 | + | 290.280813 | 483.245151 | 598.647379 |
| AKI86174.1 | 4726 | 4923 | + | 380.503997 | 509.251886 | 595.560177 |
| AKI86175.1 | 5012 | 5317 | + | 227.081932 | 276.594709 | 369.090564 |
| AKI86176.1 | 5360 | 5929 | - | 59.580154 | 70.8879294 | 76.8550955 |
| AKI86177.1 | 5922 | 6098 | - | 258.421948 | 305.857986 | 309.441948 |
| AKI86178.1 | 6192 | 6815 | - | 95.5260839 | 111.785696 | 110.868887 |
| AKI86179.1 | 6819 | 7106 | - | 66.2728387 | 83.5243234 | 86.2782442 |
| AKI86180.1 | 7108 | 7326 | - | 65.1947702 | 78.0128697 | 70.3944119 |
| AKI86181.1 | 7328 | 7543 | - | 46.7706752 | 64.7813925 | 56.5213931 |
| AKI86182.1 | 7503 | 8009 | - | 30.2933538 | 39.3830897 | 35.4119414 |
| AKI86183.1 | 8011 | 8958 | - | 36.9245622 | 51.4676231 | 42.4731124 |
| AKI86184.1 | 8955 | 9194 | - | 31.531374 | 36.4668288 | 34.5112977 |
| AKI86185.1 | 9187 | 9390 | - | 39.6540606 | 45.9849409 | 46.2341084 |
| AKI86186.1 | 9387 | 10265 | - | 50.1287299 | 65.4645571 | 57.1908896 |
| AKI86187.1 | 10373 | 10816 | - | 95.6730148 | 119.923282 | 88.5831309 |
| AKI86188.1 | 10893 | 11714 | - | 289.407016 | 349.510303 | 346.408916 |
| AKI86189.1 | 11778 | 12125 | - | 470.587145 | 572.264916 | 548.521203 |
| AKI86190.1 | 12201 | 12788 | - | 195.933872 | 234.719281 | 243.700163 |
| AKI86191.1 | 12788 | 13477 | - | 44.9772866 | 48.4198976 | 49.0218282 |
| AKI86192.1 | 13474 | 14424 | - | 47.1174795 | 58.855019 | 56.4102173 |
| AKI86193.1 | 14443 | 14724 | - | 139.595979 | 153.877101 | 134.293132 |
| AKI86194.1 | 14745 | 15026 | - | 248.126566 | 289.541696 | 264.341855 |
| AKI86195.1 | 15038 | 15250 | - | 110.785317 | 118.593314 | 116.657908 |
| AKI86196.1 | 15321 | 15968 | - | 92.9085368 | 73.3542109 | 81.4203598 |
| AKI86197.1 | 16829 | 17782 | - | 72.6226832 | 75.0678591 | 92.4918335 |
| AKI86198.1 | 17779 | 19248 | - | 49.4637137 | 58.5394431 | 108.390797 |
| **AKI86199.1 (CI)** | **19343** | **20056** | **-** | **97.373039** | **142.358807** | **209.411342** |
| **AKI86200.1 (Cro)** | **20152** | **20355** | **+** | **71.1762978** | **66.5368697** | **73.340908** |
| AKI86201.1 | 20386 | 20679 | + | 77.0294952 | 71.3026104 | 81.8304923 |
| AKI86202.1 | 20887 | 21264 | + | 71.7955752 | 82.285193 | 75.8684918 |
| AKI86203.1 | 21258 | 22778 | + | 47.4988023 | 50.3917487 | 52.4096733 |
| AKI86204.1 | 22768 | 23739 | + | 30.1641132 | 40.0064862 | 40.8825763 |
| AKI86205.1 | 23739 | 24188 | + | 26.4677149 | 28.9987716 | 35.5353323 |
| AKI86206.1 | 24193 | 24759 | + | 24.3920865 | 34.5685618 | 32.5934756 |
| AKI86207.1 | 24756 | 24950 | + | 25.9993444 | 33.8632551 | 32.163486 |
| AKI86208.1 | 24943 | 25377 | + | 15.5112722 | 15.5415104 | 21.2419548 |
| **Protein_ID** | **Start** | **End** | **Strand** | **RPMK normalized countings** | | |
|  |  |  |  | **1** | **2** | **3** |
| AKI86209.1 | 25626 | 25778 | + | 184.930458 | 194.900792 | 190.256094 |
| AKI86210.1 | 26161 | 27120 | + | 131.095959 | 158.259486 | 153.281031 |
| AKI86211.1 | 27132 | 27401 | + | 255.288497 | 325.314199 | 255.077722 |
| AKI86212.1 | 27887 | 29824 | + | 112.297068 | 123.600021 | 134.959623 |
| AKI86213.1 | 29821 | 29946 | + | 72.4859173 | 98.8531468 | 85.1145685 |
| AKI86214.1 | 29934 | 30047 | + | 198.655014 | 228.861052 | 222.235818 |
| AKI86215.1 | 30007 | 30138 | + | 350.050498 | 361.756765 | 352.246158 |
| AKI86216.1 | 30179 | 30424 | + | 285.195463 | 285.187254 | 262.090592 |
| AKI86217.1 | 30502 | 30717 | + | 44.9585273 | 48.5253876 | 50.536775 |
| AKI86218.1 | 30722 | 31255 | + | 66.4939033 | 79.9851052 | 80.2431939 |
| AKI86219.1 | 31582 | 32469 | - | 8.01822985 | 7.43618778 | 8.84213845 |
| AKI86220.1 | 32469 | 32744 | - | 12.2910903 | 10.2536254 | 9.02029394 |
| AKI86221.1 | 32852 | 33412 | + | 49.704629 | 55.3033722 | 71.6874039 |
| AKI86222.1 | 33427 | 33561 | + | 43.0773451 | 53.1838248 | 52.8419613 |
| AKI86223.1 | 33569 | 34033 | + | 42.7299472 | 45.3070587 | 56.1138385 |
| AKI86224.1 | 34065 | 34358 | - | 3193.77601 | 2316.2653 | 1652.08029 |
| AKI86225.1 | 34508 | 34711 | + | 46.4153521 | 57.5454008 | 57.7339628 |
| AKI86226.1 | 34767 | 35573 | + | 36.8396298 | 41.9519113 | 53.0977268 |
| AKI86227.1 | 35554 | 37260 | + | 23.5529184 | 25.8813438 | 30.2631654 |
| AKI86228.1 | 37260 | 39404 | + | 36.6875776 | 42.8543647 | 45.176194 |
| AKI86229.1 | 39562 | 40569 | + | 143.233652 | 148.851627 | 155.742561 |
| AKI86230.1 | 40593 | 41807 | + | 104.287675 | 115.490423 | 114.333296 |
| AKI86231.1 | 41863 | 42252 | + | 63.6601594 | 70.6828261 | 74.5161679 |
| AKI86232.1 | 42302 | 42763 | + | 68.0211075 | 68.0048646 | 77.5668683 |
| AKI86233.1 | 42747 | 43310 | + | 28.2893367 | 34.1019905 | 34.0826123 |
| AKI86234.1 | 43310 | 43960 | + | 53.5412766 | 63.1948136 | 71.7051016 |
| AKI86235.1 | 43957 | 45894 | + | 20.4089053 | 24.9056928 | 24.0002331 |
| AKI86236.1 | 45896 | 46165 | + | 56.4699813 | 56.4835512 | 58.1616218 |
| AKI86237.1 | 46251 | 46493 | + | 44.9489392 | 40.5456572 | 43.3453821 |
| AKI86238.1 | 46554 | 46673 | + | 44.4235122 | 44.1095773 | 47.0789957 |
| AKI86239.1 | 46713 | 48413 | + | 47.1624169 | 50.7899057 | 53.3232639 |
| AKI86240.1 | 48410 | 49678 | + | 17.3466805 | 20.8555921 | 21.6936511 |
| AKI86241.1 | 49693 | 49971 | + | 53.2454157 | 74.4786433 | 63.5785234 |
| AKI86242.1 | 49977 | 50594 | + | 34.0211296 | 33.4118494 | 32.3827879 |
| AKI86243.1 | 50685 | 51419 | + | 50.5668522 | 45.9901837 | 52.2412377 |
| AKI86244.1 | 51652 | 51792 | + | 2960.99464 | 4049.8669 | 3807.40554 |
| AKI86245.1 | 51849 | 52250 | + | 226.081876 | 291.434787 | 279.163081 |
| AKI86246.1 | 52344 | 53000 | + | 22.3841052 | 25.8846383 | 25.9424541 |
| AKI86247.1 | 53003 | 53449 | + | 28.8970704 | 27.9623475 | 28.7047454 |
| AKI86248.1 | 53459 | 53710 | + | 13.2397747 | 19.8607479 | 20.5186906 |
| AKI86249.1 | 53721 | 54986 | + | 25.1467732 | 27.7560618 | 28.1173052 |
| AKI86250.1 | 55056 | 63437 | + | 31.0609457 | 33.3783112 | 37.638407 |

**Table S7. Verifying the expression of *cI* and *cro* in MG1655 pWKS-*cI* and MG1655 pWKS-*cro*, respectively.** The *cI* and *cro* transcript abundance was determined by RT-PCR in total RNA isolated form the strains grown to exponential phase in the corresponding growth medium. The relative normalized expression of *cI* and *cro* was calculated using the ΔΔCt method with *gapA* being the control gene and MG1655::φO104 set as control sample, i.e. the expression of *cI* and *cro* in MG1655::φO104 was set to 1. The mean and standard deviation of three biological replicates are presented.

| **Strain** | **Growth Medium** | **Target** | **Relative Normalized Expression** |
| --- | --- | --- | --- |
| MG1655::φO104 | LB | *cI* | 1.00 ± 0.10 |
| MG1655 pWKS-*cI* | LB |  | 1.47 ± 0.28 |
| MG1655 pWKS-*cI* | MM+ 0.2% Glucose |  | 0.66 ± 0.07 |
| MG1655 pWKS | LB/MM + 0.2% Glucose |  | n.d. |
| MG1655::φO104 | LB | *cro* | 1.00 ± 0.11 |
| MG1655 pWKS-*cro* | LB |  | 3.12 ± 0.33 |
| MG1655 pWKS-*cro* | MM+ 0.2% Glucose |  | 2.73 ± 0.46 |
| MG1655 pWKS | LB/MM + 0.2% Glucose |  | n.d. |

**Fig. S9. Growth phenotypes of c*I* and *cro* expression in MG1655 in minimal medium supplemented with single carbon sources.** The strains were grown in minimal medium supplemented with 0.2% glucose, maltose, L-lactate, galactose, glycerol and ribose. The graphs represent the change in OD_595_ over time.

**
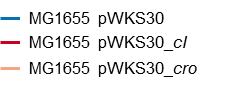
**

**Fig. S10. Heatmap of the respiration potential of MG1655 pWKS30, MG1655 pWKS30-*cI* and MG1655 pWKS30-cro.** The heatmap shows a subset of the substrates, with which the Stx2 lysogens displayed significantly reduced respiration in comparison to naïve MG1655. One biological replicates per strain was analyzed. The degree of respiration with each substrate is color coded based on the provided color key. Compare with Fig. 3 from main manuscript.

**
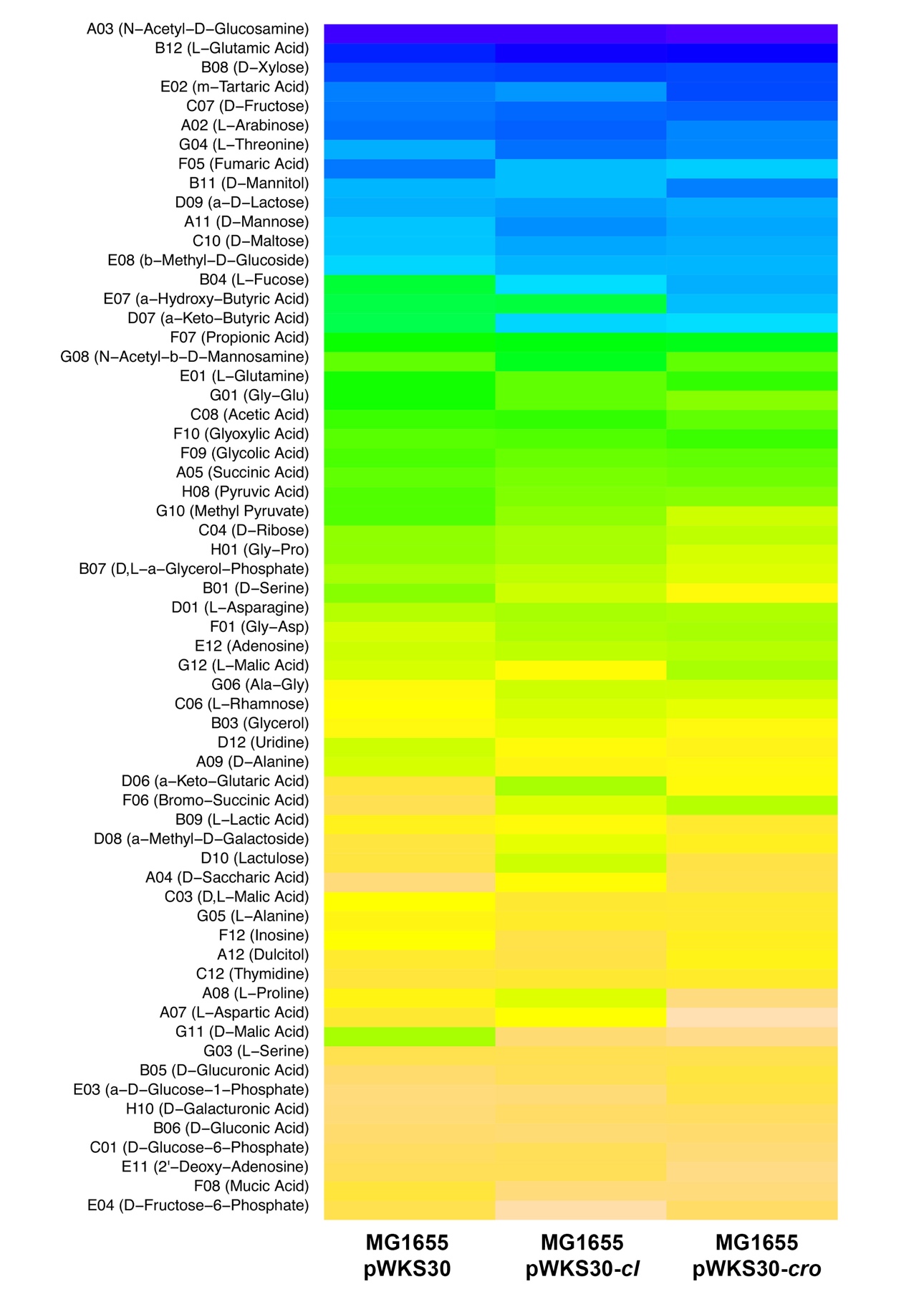
**

**
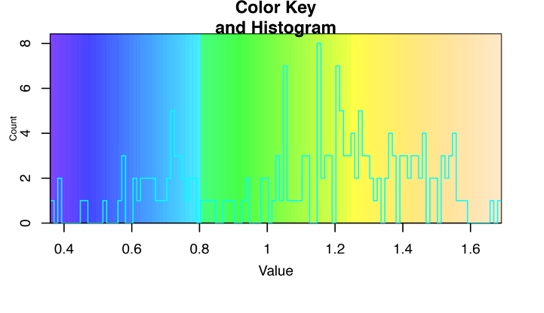
**

**Table S8. Primers used in this study.** A list of the primers and some additional information (e.g., sequence, target, reference) are provided.

| **Primer** | **Sequence (5' -3')** | **Target** | **Description** | **Reference** |
| --- | --- | --- | --- | --- |
| PB_1 | CGACCCCTCTTGAACATA | *stx2* | forward primer | Bielaszewska at al., 2012 |
| PB_2 | TAGACATCAAGCCCTCGTAT |  | reverse primer | Bielaszewska at al., 2012 |
| PB_3 | GCGGGTAGGTGTACCAAAAA | *wrbA* | forward primer | this study |
| PB_5 | CGTGACTCAAGTTGCCATGT | φO104 | reverse primer | this study |
| PB_8 | TCGTAGTTTGGTGTCGCTCA | φPA8 | forward primer | this study |
| PB_7 | GGATGGGAGCGAACTGATAA | *argW* | reverse primer | this study |
| PB_9 | GCTAACCTGAAATGGGACGA | *gapA* | forward primer | this study |
| PB_10 | AACCACTTTCTTCGCACCAG |  | reverse primer | this study |
| PB_11 | TCCTTCAAAATGCCCATATCA | -88 of *cI* ATG | forward primer | this study |
| PB_12 | AATCCTCTTTACGTGGCATGA | +90 of *cI* TAA | reverse primer | this study |
| PB_13 | CGTTTTGATTTGCCTGTTCA | -105 of *cro* ATG | forward primer | this study |
| PB_14 | TCATGTTCATACCATATCACCTTTG | +51 of *cro* TAA | reverse primer | this study |
| PB_15 | GTTAACCCCTGAGCAACTGG | c*I* | forward primer | this study |
| PB_16 | TGCGACAGGCCTAACTCTTT |  | reverse primer | this study |
| PB_17 | GCGCAGAGTGCAATCAGTAA | *cro* | forward primer | this study |
| PB_18 | CTCTTACGAACGCAGGGAAC |  | reverse primer | this study |
| M13_rev | CAGGAAACAGCTATGAC | pWKS30 | sequencing primer | universal primer |
